# Vascular smooth muscle-inspired architecture enables soft yet tough self-healing materials for durable capacitive strain-sensor

**DOI:** 10.1038/s41467-023-35810-y

**Published:** 2023-01-10

**Authors:** FuYao Sun, LongFei Liu, Tong Liu, XueBin Wang, Qi Qi, ZuSheng Hang, Kai Chen, JianHua Xu, JiaJun Fu

**Affiliations:** 1 School of Chemistry and Chemical Engineering, Nanjing University of Science and Technology, Nanjing, 210094 China; 2 Jiangsu Co-Innovation Center of Efficient Processing and Utilization of Forest Resources, College of Chemical Engineering, Nanjing Forestry University, Nanjing, 210037 China; 3 Jiangsu Key Laboratory of Advanced Structural Materials and Application Technology, Nanjing Institute of Technology, Nanjing, 211167 China; 4 State Key Laboratory of Coordination Chemistry, Nanjing University, Nanjing, 210093 China; 5 School of Science, Nanjing University of Science and Technology, Nanjing, 210094 China

**Keywords:** Composites, Polymers, Polymers

## Abstract

Catastrophically mechanical failure of soft self-healing materials is unavoidable due to their inherently poor resistance to crack propagation. Here, with a model system, i.e., soft self-healing polyurea, we present a biomimetic strategy of surpassing trade-off between soft self-healing and high fracture toughness, enabling the conversion of soft and weak into soft yet tough self-healing material. Such an achievement is inspired by vascular smooth muscles, where core-shell structured Galinstan micro-droplets are introduced through molecularly interfacial metal-coordinated assembly, resulting in an increased crack-resistant strain and fracture toughness of 12.2 and 34.9 times without sacrificing softness. The obtained fracture toughness is up to 111.16 ± 8.76 kJ/m^2^, even higher than that of Al and Zn alloys. Moreover, the resultant composite delivers fast self-healing kinetics (1 min) upon local near-infrared irradiation, and possesses ultra-high dielectric constants (~14.57), thus being able to be fabricated into sensitive and self-healing capacitive strain-sensors tolerant towards cracks potentially evolved in service.

## Abstract (web summary)

Catastrophically mechanical failure, of soft self-healing materials often stems from its poor resistance to crack, propagation. Here, the authors present a strategy of surpassing trade-off, between soft self-healing and high fracture toughness, enabling the, conversion of soft and weak into soft yet tough self-healing materials.

## Introduction

Compared with traditional heavy materials such as metals and their alloys, soft polymeric materials including elastomers and gels feature excellent mechanical compliance, high extensibility, good conformability, and light weight, thus having been growingly critical in various brand-new applications, such as wearable electronics, soft robotics and biomedical devices^1–3^. To prolong their service life and protect them from wear and tear in dynamic application environments, great efforts have been devoted to rendering soft materials self-healable without compromising their intrinsic softness. A popular strategy is incorporating weak non-covalent interactions into existing soft polymeric networks capable of recombining after damage^4–6^. However, the inherently weak bond strength of non-covalent interactions inevitably imposes an upper limit in fracture energy (a definition of toughness) around ≈0.1–1.0 kJ m^−2^ or less. This limitation causes soft self-healing materials easily start to break at the site of microcrack exposed during deformation^7,8^, thus severely reducing or even depriving the operation reliability of their integrated stretchable devices. To address this limitation, double cross-link strategies based on two kinds of non-covalent interactions with different binding strengths have been implemented, in which the rupture of relatively weak interactions can realize reversible energy dissipation^8,9^. Nonetheless, it still does not lead to a qualitative leap in terms of fracture energy. As predicted by Lake and Thomas, the threshold fracture energy (minimum energy necessary to break elastomeric network) of a covalent elastomer scales with *J*, where *J* is chemical energy per covalent bond^10,11^. Consequently, the stronger the bond strength, the tougher the elastomer becomes in theory; however, there is always a trade-off between the self-healing ability and bond strength^12,13^. To circumvent this contradiction, soft self-healing polymers have been toughened by introducing permanent molecular-scale covalent crosslinks into reversible networks^8,14^, by incorporating stiff yet incompatible polymers/oligomers to form nano-/micro-scale phase separation structure^15,16^, or by embedding meso-/macro-scale rigid fillers into polymer matrix to transfer local stress^17,18^. The optimizations of networks, structures or fillers at different scales are therefore expected to dramatically enhance fracture energy through increasing mechanical dissipation in regions around crack, but always at the cost of intrinsic softness (Supplementary Fig. 1).

**Fig. 1:**
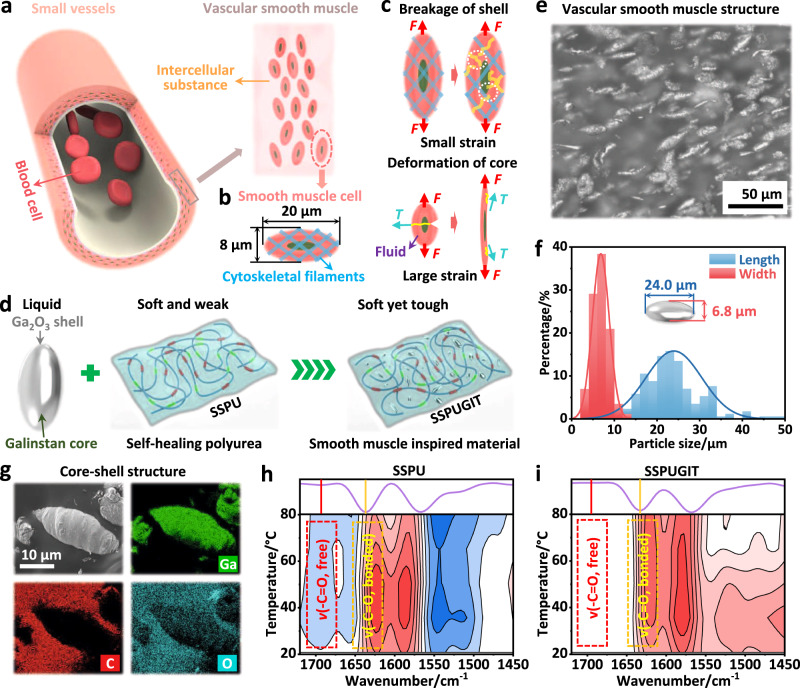
Structural design of the SSPUGIT composite. Schematic illustration of (**a**) a vascular smooth muscle structure and **b** the corresponding structure and size of a single vascular smooth muscle cell. **c** Schematic illustration of multimodal toughening mechanism of vascular smooth muscle cell in pure shear process; *F* represents the tensile stress, while *T* represents the shear stress in loading. **d** Schematic fabrication process of vascular smooth muscle-inspired SSPUGIT composite. **e** Optical microscopy image of SSPUGIT composite. **f** Size distribution of Galinstan micro-droplets in the SSPUGIT composite from **e**. **g** Scanning electron microscopy (SEM) and the corresponding elemental mapping images of the SSPUGIT composite. PCMW synchronous spectrum of **h** SSPU and **i** SSPUGIT calculated from temperature-dependent 2D-FTIR spectra. The red color represents positive spectral intensity, while blue color represents negative ones.

Our strategy is inspired by the soft yet tough smooth muscle tissue of animal’s small vessels that can withstand drastic changes in blood pressure during continuous contraction and relaxation^19^. Vascular smooth muscles compose of spindle-like smooth muscle cells (major axis: ~20 μm, minor axis: ~8 μm) dispersed in intercellular substance (Fig. 1a)^20^. The muscle cells act as the soft toughened units resisting crack growth to offer remarkable defect tolerance and high fracture toughness. In essence, smooth muscle cells typically have core-shell structures^21^, in which their thin outer shell is enclosed by cytoskeletal filaments of relatively high strength, while the large inner core contains mostly fluid viscous substances (Fig. 1b). According to the multimodal toughening mechanism, the breakage of outer cytoskeletal network at small strain increases mechanical energy dissipation near the crack tip^22^; and meanwhile, the intrinsic liquid character of inner core contributes to blunting, deflecting or even vertically eliminating the crack during crack propagation at large strain (Fig. 1c). Since the fluid viscous substances have almost zero modulus^23^, it preserves the intrinsic softness of vascular smooth muscles. Altogether, such a unique structure allows vascular smooth muscles to integrate excellent compliance and high fracture toughness. Inspired by this natural design concept, we are motivated to mimic the structure of vascular smooth muscles to tremendously increase the fracture energy/toughness of soft self-healing polymers while retaining their softness to the greatest extent. However, such a biomimetic self-healing material has not been developed so far due to the extreme challenge of assembling liquid micro-droplets within soft self-healing polymer matrixes, which necessitates good compatibility, dispersibility and miscibility.

Here, we report our successful attempt that a class of core-shell structured liquid droplets, i.e., gallium–indium–tin eutectic alloys (Galinstan) wrapped with thin oxide layer^24,25^, can be purposely introduced into a soft self-healing polyurea (SSPU) without settling or agglomeration through robust interfacial coordination interactions (Fig. 1d; Supplementary Fig. 12), leading to a synthetic structure similar to that of vascular smooth muscles (Fig. 1e). The SSPU system we used to illustrate this strategy is based on dynamic hard domains concept that our laboratory had previously demonstrated for constructing soft self-healing polyureas^26,27^. As expected, soft Galinstan droplet acts like vascular smooth muscle cells that can deform with SSPU matrix along the tensile direction, causing the transverse crack to branch and then longitudinally deflect to propagation until fracture. Such an autonomous crack elimination behavior allows the biomimetic SSPU/Galinstan (SSPUGIT) material to tremendously increase mechanical dissipation compared to traditional unfilled polymers or rigid-filler-containing composites, leading to the unprecedented enhancement of defect-tolerant performances. Compared to the initial SSPU, the biomimetic SSPUGIT material exhibited significant improvement in fracture toughness and crack-resistant strain by up to 34.9 and 12.2 times, respectively, while the increase of Young’s modulus was inappreciable. Specifically, the SSPUGIT composite was completely insensitive to crack propagation, and possessed an extremely high fracture toughness of 111.16 ± 8.76 kJ m^−2^, which far exceeds the highest value for reported soft self-healing material, and even higher than that of Al and Zn alloys. More interestingly, assisted with the outstanding photothermal properties of vascular smooth muscle-inspired architecture assembled from Galinstan droplets under near-infrared (NIR) laser irradiation, an ultra-fast self-healing with a high healing efficiency of ~98.13% ± 1.93% was accomplished within 1 min. Our work represents an exciting and straightforward bioinspired strategy for the mass production of soft yet tough self-healing materials. The methodologies developed here showed great potential to be applied to various liquid fillers/polymer systems based on other chemistries, inspiring the design of soft self-healing materials with extremely enhanced fracture toughness for a wide range of stretchable and functional devices.

## Results

### Material design and fabrication strategy

Aiming to demonstrate our bionic strategy of extremely toughening soft self-healing materials without jeopardizing their softness, a model SSPU was first synthesized by a one-pot polycondensation reaction between bis(3-aminopropyl)-terminated poly(dimethylsiloxane) (PDMS, M_n_ = 3000) and a mixture of trimethyl hexamethylene diisocyanate (THI) and 3,3′-dimethyl-4,4′-biphenylene diisocyanate (DBI) (molar ratio, PDMS:THI:DBI = 10:6:4). Detailed polymerization procedures of SSPU and their chemical composition analysis were included in supporting information (Supplementary Figs. 2–4). Our core strategy to simultaneously achieve softness and self-healing is based on the irregular self-sorting of trimethyl hexamethylene-urea (THU) and 3,3′-dimethyl-4,4′-biphenylene urea (DBU) units at a molecular level, which contributes to a highly dynamic hard domain with amorphous structure constructed by multiple hydrogen bonds (Supplementary Figs. 5–7)^26,27^. Meanwhile, the abundant urea motifs in SSPU network are capable of forming robust coordination interactions with the gallium oxide shell of Galinstan^28^, which is highly desired for the uniform dispersion of liquid Galinstan into SSPU matrix, towards a dramatic enhancement of comprehensive mechanical properties, especially the crack-resistant strain and facture toughness.

With the exquisitely designed soft self-healing polyurea, we proceeded to fabricate vascular smooth muscle-inspired SSPUGIT composite through a hierarchical structure design at multiscale levels. Specifically, micro-scale Galinstan droplets were embedded into a SSPU matrix through coordination bonding self-assembly at molecular level (Fig. 1d). A monolithic sample with dimensions of 220 × 200 × 0.5 mm could be successfully fabricated, reflecting the high scalability of this technological process (Supplementary Fig. 8). Attenuated total reflection FTIR spectroscopy was employed to analyze the compositions of the obtained SSPUGIT (Supplementary Fig. 9). The structure of SSPUGIT was characterized by optical microscopy (Fig. 1e). As observed, spindle Galinstan micro-droplets were uniformly dispersed in SSPUGIT to form a unique hierarchical structure instead of macro-phase separation. Such a spindle shape was similar to that of the vascular smooth muscle cell, which was attributed to the rotational orientation of Galinstan micro-droplets during continuous stirring (Supplementary Fig. 10). More interestingly, the average sizes of the spindle micro-droplets in SSPUGIT are very close to that of the vascular smooth muscle cell, i.e, their major axis and minor axis are around 24.0 μm and 6.8 μm, respectively (Fig. 1f). In addition, as depicted by energy dispersive spectroscopy (EDS) mapping images, the abundant O element on the surface of Galinstan micro-droplets confirmed the formation of thin oxide shell (Fig. 1g), which is similar to the role of outer cytoskeletal network in vascular smooth muscle cell. For better comparison, a control sample (SSPUGIT-C) was fabricated by extrusion combined with hot pressing method. Since the shearing process during extrusion can break the vascular smooth muscle-like structure (Supplementary Fig. 11)^29^, SSPUGIT-C is an ideal model sample to demonstrate the particularity of structure on comprehensive mechanical performance.

The chemical structure of the robust interfacial coordination interaction between Galinstan and SSPU at molecular level is shown in Supplementary Fig. 12, and further confirmed by temperature-dependent FT-IR spectra and UV–vis absorption spectra (Supplementary Figs. 13–14). Perturbation correlation moving window (PCMW) technology was employed to convert the temperature-dependent FTIR spectra, aiming at visualizing the subtle variations of the different motifs at a high resolution^30^. Figure 1h, i shows the synchronous PCMW spectra of SSPU and SSPUGIT, respectively. For SSPU, two correlation cross peaks centered at 1634 cm^−1^ (positive correlation, hydrogen-bonded –C = O motifs, Supplementary Fig. 13) and 1692 cm^−1^ (negative correlation, free –C = O motifs, Supplementary Fig. 13) are evolved in the temperature range of 20–80 °C (Fig. 1h). These variations demonstrated that the hydrogen bonding between different urea motifs gradually broke with temperature increasing^31^. However, as for SSPUGIT composite, the negative correlation peak of free –C = O motifs disappeared during the whole heating process (Fig. 1i). These results suggested that there were no free –C = O motifs generated upon heating, which could be attributed to the formation of robust coordination interaction between –C = O motifs and gallium oxide shell^32^. This idea was further verified by UV–vis absorption spectra, in which the characteristic peak of Galinstan arising from plasmon resonance exhibited a distinct red shift feature after complexing with SSPU (Supplementary Fig. 14)^33^. Such robust and abundant interfacial coordination interactions could facilitate the uniform dispersion of Galinstan droplets in SSPUGIT composite^25^, thus improving the compatibility and miscibility between Galinstan and SSPU matrix to prevent macro-phase separation (Supplementary Fig. 8). As the concentration of –C = O motifs was significantly decreased in SSPU network, Galinstan droplets would agglomerate and settle to form macro-phase separation (Supplementary Fig. 15), demonstrating the key role of interfacial coordination interactions to the successful imitation of vascular smooth muscle structure.

### Soft yet tough performance

**Fig. 2:**
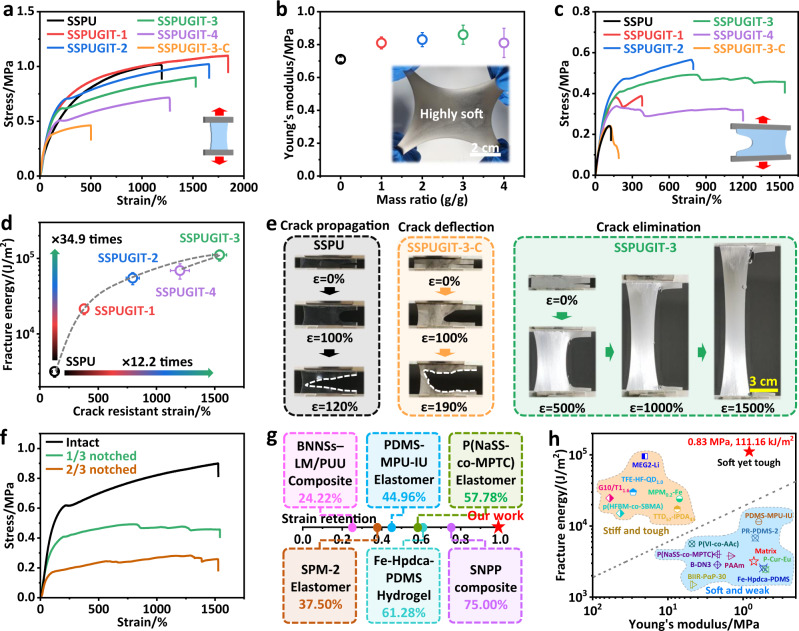
Soft yet tough SSPUGIT composite. **a** Stress-strain curves of SSPU, SSPUGIT-3-C, and SSPUGIT composites with different Galinstan content from 100 wt% to 400 wt%. **b** Comparison on the Young’s modulus of the SSPU and SSPUGIT composites, inserted images showing high softness of SSPUGIT composites. Error bars represent the standard deviation calculated by the data sets (*n* = 3). **c** Stress-strain curves of SSPU, SSPUGIT-3-C, and SSPUGIT composites by pure shear test. **d** Fracture toughness and crack-resistant strain of SSPU and SSPUGIT composites. Error bars represent the standard deviation calculated by the data sets (*n* = 3). **e** Photographs of the notched SSPU, SSPUGIT-3-C, and SSPUGIT-3 composites being elongated to different strains. **f** Stress-strain curves of the intact and notched SSPUGIT-3 with notch ratios of 1/3 and 2/3. **g** Comparison of the strain retentions of SSPUGIT-3 with reported crack-resistant self-healing materials reported previously. **h** Comparison of the fracture toughness versus Young’s modulus among state-of-the-art self-healing materials with SSPUGIT-3. Our work demonstrates high fracture toughness with low Young’s modulus. The gray dashed line indicates the performance upper bound among the state-of-the-art elastic materials defined by Zhang and co-workers^42^.

Due to the uniquely bionic architecture of SSPUGIT composites, their mechanical performances, especially the crack resistance and fracture toughness, had been effectively improved with intrinsic softness retained. The fracture toughness (Г) of SSPU and SSPUGIT-X (X is the mass ratio between Galinstan and SSPU) were measured using the widely adopted Rivlin-Thomas single-notch method (Supplementary Fig. 16)^34^. The Young’s modulus (E) was recorded to evaluate the stiffness or softness, which was obtained from the slope of the initial parts in stress-strain curves (Supplementary Fig. 17). As shown in Fig. 2a, the tensile curve of SSPU exhibited a typical pattern of elastomer, showing a relatively low Young’s modulus of 0.72 ± 0.02 MPa (Fig. 2b) comparable to that of other soft self-healing materials^35,36^. However, the crack resistance and fracture toughness of SSPU were very poor. Once a crack existed in SSPU, its fracture strain would sharply decrease due to the continuous horizontal propagation of the crack (Fig. 2c, e, Supplementary Movie 1). Specifically, the fracture strain of the intact SSPU sample was up to 1193.29 ± 12.45% (Supplementary Fig. 16), while it inconceivably dropped to 121.66 ± 21.24% for the notched sample (Fig. 2c), corresponding to an ultralow fracture toughness of 3.18 ± 0.51 kJ m^−2^. In sharp contrast, SSPUGIT composites exhibited a distinguished combination of crack resistance and fracture toughness. As the Galinstan content increased from 100 wt% to 400 wt%, the crack-resistant strain increased from 380.21 ± 34.17% to 1541.75 ± 61.32% and then decreased to 1201.48 ± 81.29%; the fracture toughness increased from 21.51 ± 3.58 to 111.16 ± 8.76 and then decreased to 69.05 ± 15.47 kJ m^-2^ (Fig. 2d; Supplementary Fig. 16). However, the tensile stress and tensile strain of SSPUGIT composites gradually dropped as Galinstan content increased (Fig. 2a), which was consistent with the phenomenon found by Haque and co-workers^37^. These reductions are in line with the classical linear elastic fracture mechanics (LEFM) theory that composites with much more filler droplets will break at lower stress (and hence lower strain) than composites with fewer droplets^38^. To sum up, the toughening effect and mechanical properties of the vascular smooth muscle-like structure on SSPUGIT composites are heavily dependent on the Galinstan content.

Intriguingly, given the mechanical biomimetic strategy, the SSPUGIT composites retained a low Young’s modulus despite their dramatically increased fracture toughness, which is highly desired for compliant and deformable devices that is difficult to achieve^39,40^. As shown in Fig. 2b, after the incorporation of vast Galinstan liquid drops, the fabricated composites were still highly soft, with a low tensile modulus close to that of SSPU matrix. Such a negligible increase in modulus or stiffness may be attributed to the stronger influence of rigid Ga_2_O_3_ oxide layer on stress, and interphases formed between SSPU and Galinstan^23^. The optimal Galinstan content for SSPUGIT composites was 300 wt%, i.e., SSPUGIT-3, which showed a soft yet tough characteristic with the best combination of high crack-resistant strain (1541.75 ± 61.32%), high fracture toughness (111.16 ± 8.76 kJ m^−2^), and low Young’s modulus (0.83 ± 0.05 MPa). Compared to the initial SSPU matrix, the improvements of crack-resistant strain and fracture toughness for SSPUGIT-3 are approximately up to 12.2 and 34.9 times, respectively (Fig. 2d). These huge enhancements were also visually illustrated in Fig. 2e, where SSPUGIT-3 presented a remarkable crack-elimination ability that the pre-existing crack gradually widened along the tensile direction but hardly advanced into materials even at high strains (Supplementary Movie 3). However, SSPUGIT-3-C control sample subjected to the shearing process showed lower mechanical enhancements, i.e., its crack-resistant strain and fracture toughness were only 190.73 ± 15.34% and 5.83 ± 0.45 kJ m^−2^ (Fig. 2c; Supplementary Movie 2), respectively, much lower than those of SSPUGIT-3. Certainly, the particularity of microstructure is the core factor related to the toughening effect (Fig. 1e; Supplementary Fig. 11). SSPUGIT-3 featured an optimized structure working on a multimodal toughening mechanism similar to vascular smooth muscle, which had the ability to efficiently deflect or even eliminate crack with enormous energy dissipation, resulting in the significant improvement of fracture toughness for composites.

For most soft self-healing elastomers, the fracture strain of the notched samples is always smaller than that of the intact sample even though they are crack-resistant. Typically, a tough self-healing polymer, reported by Bao and co-workers, showed a crack-resistant stretching up to ~780%, which is only ~45% of the fracture strain for the intact sample (~1730%)^8^. However, as depicted by the pure shear test of SSPUGIT-3 (the size of notch is a third of the whole width), the notched sample possessed almost the same fracture strain as the intact sample (Fig. 2f; Supplementary Fig. 15). Moreover, when we proceeded to increase the proportion of notch to two third of the width, the fracture strain retention of the notched SSPUGIT-3 was still 100% (Fig. 2f; Supplementary Fig. 18 and Movie 3). These results suggest our SSPUGIT-3 is completely insensitive to crack in service, far superior to self-healing materials ever reported to date (Fig. 2g; Supplementary Table 1). Generally, low fracture toughness hinders the long-term usability of self-healing materials, especially if they are exposed to mechanical damages^41^. Therefore, we compared the Young’s modulus and fracture toughness of SSPUGIT-3 with other self-healing materials, as showed in Fig. 2h where the gray dashed line (Γ = 18915 kN^3/2^m^−2^ × E^−1/2^) indicated an upper bound of fracture toughness to Young’s modulus among the state-of-the-art elastic materials proposed by Zhang and co-workers^42^. Our SSPUGIT-3 material broke through the limited constraint of the statistical formula, exhibiting an extremely high fracture toughness up to 111.16 ± 8.76 kJ m^−2^ (which is even higher than that of Al and Zn alloys), together with a low Young’s modulus of 0.83 ± 0.05 MPa (Fig. 2h; Supplementary Table S2). Such soft yet tough property position our bionic composite in a previously unexplored area of performance in self-healing materials, and offer an effective way for known trade-off in soft self-healing and fracture toughness.

### Mechanisms of crack resistance

**Fig. 3:**
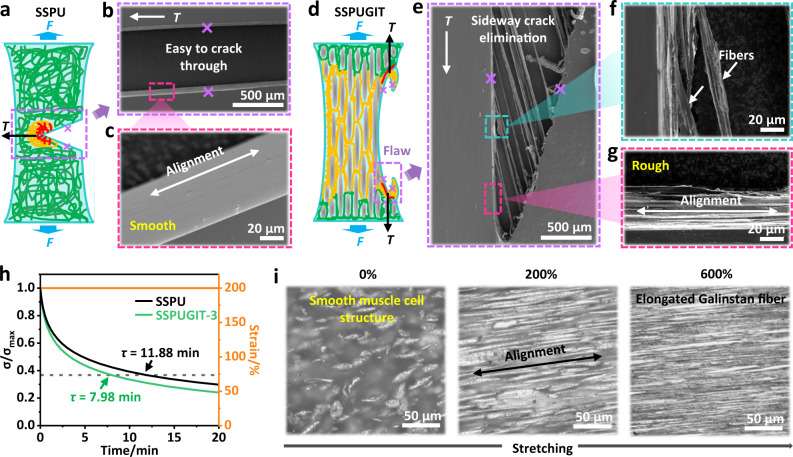
Mechanisms analysis for fracture process of the SSPUGIT composite. **a** Schematic illustration of stress distribution for SSPU in a pure shear test; red, orange, and green lines represent high, middle and low stress, respectively. **b** SEM images of the fracture areas in **a**, showing straight crack propagation perpendicular to the stretching direction; *T* represents the local shear stress during the stretching process. **c** Alignment of polymer chains in a smooth facture interface of SSPPU. **d** Schematic illustration of stress distribution for SSPUGIT-3 in pure shear test. **e** SEM images of the facture areas in **d**, exhibiting sideway crack elimination parallel with stretching direction. **f** Amplified SEM images of **e** showing the fibril structure in SSPUGIT-3. **g** Alignment of fibers in a rough fracture interface of SSPPUGIT-3. **h** Stress relaxation curves indicating more mechanical energy dissipation of SSPUGIT than SSPU to 200% strain. **i** Optical microscopy images of SSPUGIT-3 at different strains (0, 200, and 600%).

To investigate the fracture behavior and toughening mechanism of SSPUGIT composite, we monitored the crack evolution around the crack tip in pre-notched specimens upon loading by SEM (Fig. 3a–g). For notched SSPU, its crack was easy to propagate promptly through the entire sample at horizontal direction (Figs. 2e and 3b; Supplementary Movie 1), resulting in a smooth fractural surface with polymer chains aligned perpendicular to the stretching direction (Fig. 3c). Contrarily, the advancing crack in the notched SSPUGIT composite gradually opened up and blunted, or even deflected at the beginning of stretching (Figs. 2e and 3d), similar to the crack-resistant vascular smooth muscle and the synthetic materials with preferentially aligned structures, that are, nacre-mimic or tendon-inspired architectures^43–45^. Once the tensile strain further increased, the crack tip branched and generated two flaws (Supplementary Fig. 19), which tended to longitudinally move along the stretching direction until they reached the upper and lower ends of the SSPUGIT composite (labeled by a purple box in Fig. 3d; Supplementary Movie 3). Moreover, SEM images of flaws of notched SSPUGIT revealed distinct fracture behaviors (Fig. 3e), where characteristic fibrillar delamination is along the tensile direction (Fig. 3f), resulting in a rough interface with fibril alignment (Fig. 3g). Such adaptive flaw movements during pre-notched SSPUGIT stretching enabled a unique sideway crack elimination that alleviated the local stress concentration around crack tip (Fig. 3d), thus allowing the notched SSPUGIT stretch to its original strain limit with a high fracture toughness (Fig. 2c, f).

Despite all these findings, it is necessary to profoundly understand why the transverse pre-crack in SSPUGIT can bifurcate and then redirect along the stretching direction in pure shear test, especially why the fibrillar delamination formed after longitudinal crack propagation. Thus, we first focus on the variations of stress-strain curves for SSPU and SSPUGIT (Fig. 2a). As depicted, a distinct yield point emerged at strain around ~200% for SSPUGIT composites, which was mainly attributed to the breakage of outer Ga_2_O_3_ shell and rupture of the interfacial coordination interactions between Ga_2_O_3_ and SSPU matrix^46^. Remarkably, these mechanical behaviors at relatively small strain (≤200%) contribute to more energy dissipation (Fig. 3h; Supplementary Fig. 20)^47,48^, permitting the deflection and branching of crack tip to diminish the stress concentration (Supplementary Fig. 21). With the increase of tensile strain, the aligned liquid inner core was further elongated into long and thin fiber accordingly^49^, as confirmed by optical microscopy images in stretching (Fig. 3i). For the highly stretched SSPUGIT up to 600% strain, there were obvious interfaces between elongated liquid Galinstan and SSPU elastomer. However, their interfacial binding energy is extremely low (approximately zero) due to the ultra-high surface tension of the shell-bursted Galinstan (600 mN m^−1^)^50^, which hinders Galinstan from wetting SSPU. According to Cook-Gordan crack-stopping mechanism^51^, if the interfacial binding energy between Galinstan and SSPU was lower than the cohesion energy of SSPU solid, the interface would be opened upon loading, vertical cracks subsequently initiated (flaws in Fig. 3d) and propagating along it (Supplementary Fig. 21). Such vertical cracks sometimes broke an elongated Galinstan fiber, which then transmitted to the adjacent one and continue to move longitudinally (Supplementary Movie 3). Eventually, the primary notch was totally eliminated to generate typical fibrillar delamination behavior (Fig. 3e), thus resulting in the large enhancement of crack-resistant strain and fracture toughness for SSPUGIT^52^.

### NIR-induced fast self-healing

**Fig. 4:**
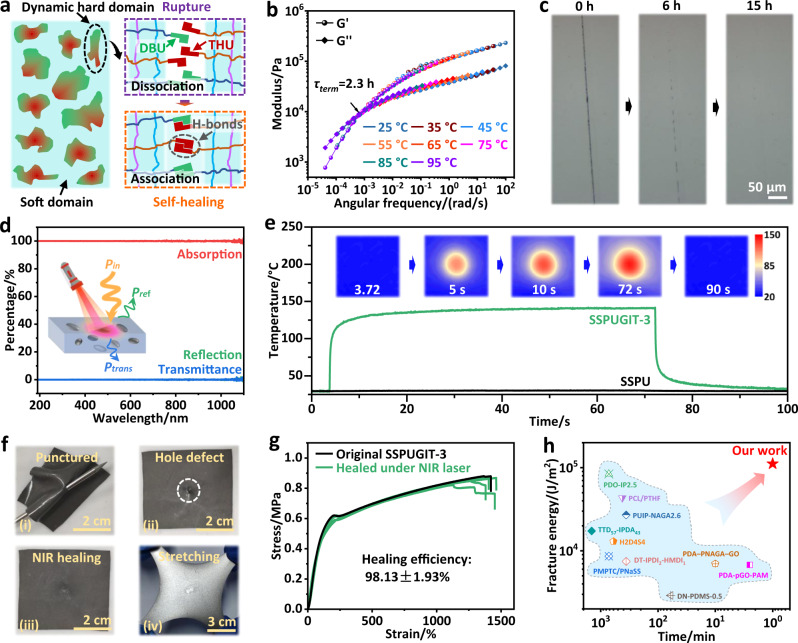
Self-healing performance and mechanism of the SSPUGIT composite. **a** Schematic illustration of the proposed mechanisms behind the self-healing process of SSPU. **b** Master curves of frequency dependence of storage modulus (G’) and loss modulus (G”) for SSPU. **c** Optical microscopic images of the scratching-healing process of SSPU at 25 °C. **d** UV-vis-NIR light absorption, reflection and transmittance spectra of SSPUGIT-3. **e** Time-temperature FLIR images and the corresponding photothermal curves of SSPU and SSPUGIT-3 irradiated at 1.99 W cm^−2^. **f** Puncture damage of SSPUGIT-3 film by a needle was healed by NIR irradiation within 1 min and then stretched without rupture. **g** Stress-strain curves of the original and healed SSPUGIT-3 samples. **h** Comparison of the healing time and healed fracture toughness among state-of-the-art soft self-healing materials with SSPUGIT-3. Our materials demonstrating ultra-high fracture toughness with short healing time.

The SSPU material possesses a distinct microphase-separated structure (Fig.4a; Supplementary Fig. 5), in which the amorphous hard domains are supramolecular aggregates assembled by multiple hydrogen bonds derived from DBU and THU units (Supplementary Figs. 6–7), while the soft domains consist of abundant PDMS polymer chains. Own to the highly dynamic nature of the constructed hard domains^26,27^, SSPU is expected to achieve autonomous self-healing at room temperature. Rheological master curves of SSPU were first fitted to study the chain dynamics, which are generally considered as the core factors determining healing property^53,54^. As depicted in Fig. 4b, the terminal relaxation time (*τ*_term_) calculated by the 2π/*ω*_term_ formula was about 2.3 h for SSPU chains at 25 °C, comparable to those reported in other self-healing systems^27,55^. Such a relatively low *τ*_term_ value implies good room-temperature self-healing kinetics of SSPU, which was further verified by dynamic thermomechanical analysis (DMA) (Supplementary Fig. 22). Scratch-healing test was then conducted to demonstrate the self-healing performance of SSPU, in which a visible artificial scratch faded away upon autonomously healing for 15 h at room temperature (Fig. 4c). Furthermore, longer healing time of 24 h was required to fully restore its mechanical properties (Supplementary Fig. 23). However, such low healing rates would cause the materials to lose their functionalities during healing process, thereby hindering the use of self-healing materials in many applications^56^. According to the time-temperature equivalence principle (Supplementary Fig. 24)^57^, increasing of temperature can enhance chain dynamics to enable a higher self-healing rate (Supplementary Fig. 25), but this is always inaccessible during most of the real-world applications, especially for the all-in-one stretchable devices intolerant of high temperature. By comparison, remotely and instantly light stimuli can straightway process the damaged regions with high accuracy, not interfering with other irrelevant areas^58^.

Exhilaratingly, our SSPUGIT composites showed excellent near-infrared (NIR)-absorbing ability to realize efficient NIR light-to-heat conversion, which may be caused by the localized surface plasmon resonance of the core-shell structured Galinstan droplets^59^. As depicted in Fig. 4d, the UV-vis-NIR absorption spectra of SSPUGIT-3 composite exhibited ultrahigh light absorptions up to ~99% around 808 nm, indicating its great potential of light-to-heat conversion to rapid and precise NIR-triggered healing. To confirm this, SSPUGIT-3 sample was irradiated by an intensity-modulated NIR laser (*λ* = 808 nm), and the visualized photothermal conversion process was recorded with an infrared thermal camera (Supplementary Fig. 26). As given in Fig. 4e, the surface temperature of SSPUGIT-3 was quickly increased in a few seconds (2 s) upon irradiation at 1.99 W cm^−2^, together with a local temperature reaching up to a high level (117.1 °C). In sharp contrast, the pristine SSPU represented no evident temperature increase at the same experimental conditions (Fig. 4e). These results illustrate that our bionic structure assembled by Galinstan droplets possesses terrific photothermal conversion capability, contributing to the fast self-healing with high accuracy. Moreover, we proceed to quantify the light-to-heat conversion rates of SSPUGIT-3 at different laser powers, in which the final surface temperature can be controllably regulated from 70 to 210 °C relying on the laser power (Supplementary Fig. 27). Given that the terminal relaxation temperature for SSPUGIT-3 was around 102 °C (Supplementary Fig. 28), thus we set a suitable laser power of 1.99 W cm^−2^ to trigger fast self-healing.

For soft and stretchable electronics, puncture damages including hole or point defects are problematic because a small defect can induce significant stress concentration on being deformed, thus causing the whole device to break easily with function failure. In consideration of this, a free-standing SSPUGIT-3 film (~300 μm) was punctured by a needle to produce a hole defect, which was then healed with NIR light for <1 min at 1.99 W cm^−2^ (Fig. 4f). We noted that the punctured hole was fully restored and did not break even if stretched, while an inconspicuous healing trail was still visible (Fig. 4f; Supplementary Fig. 29). Subsequently, we evaluated the NIR healing of total cut damages, where the SSPUGIT-3 composite was completely cut into two separate pieces and then brought into gentle contact for healing assessment (Fig. 4g). The healing efficiency (η) was defined as the recovery of integral area under stress-strain curves, which simultaneously took the restoration of both stress and strain into consideration^60^. The cut-damaged SSPUGIT-3 film almost fully restored its mechanical properties (including Young’s modulus, tensile stress, and strain) upon NIR irradiation within 1 min, approaching a high healing efficiency of 98.13% ± 1.93% (Fig. 4g), which is superior to self-healing at room temperature and high temperature (Supplementary Fig. 30). This outstanding healing performance was also visually confirmed in Supplementary Fig. 31 that the SSPUGIT-3 did not rupture at the healed portion but the non-healed areas. In general, our SSPUGIT-3 composite had a fracture toughness up to 111.16 ± 8.76 kJ m^−2^ after an optimal healing for 1 min. To the best of our knowledge, such a fast self-healing rate with ultra-high toughness is much better than the reported to date for soft self-healing materials (Fig. 4h; Supplementary Table 3). Not limited to self-healing, our SSPUGIT composites also possess excellent solvent-reprocessibility and recyclability (Supplementary Fig. 32), which are highly desired for recycling waste offcuts. Moreover, the Galinstan droplets within SSPUGIT matrix can be recycled in a high efficiency as well, which can be reused for the fabrication of new composite successfully (Supplementary Fig. 33).

### Capacitive strain-sensors application

**Fig. 5:**
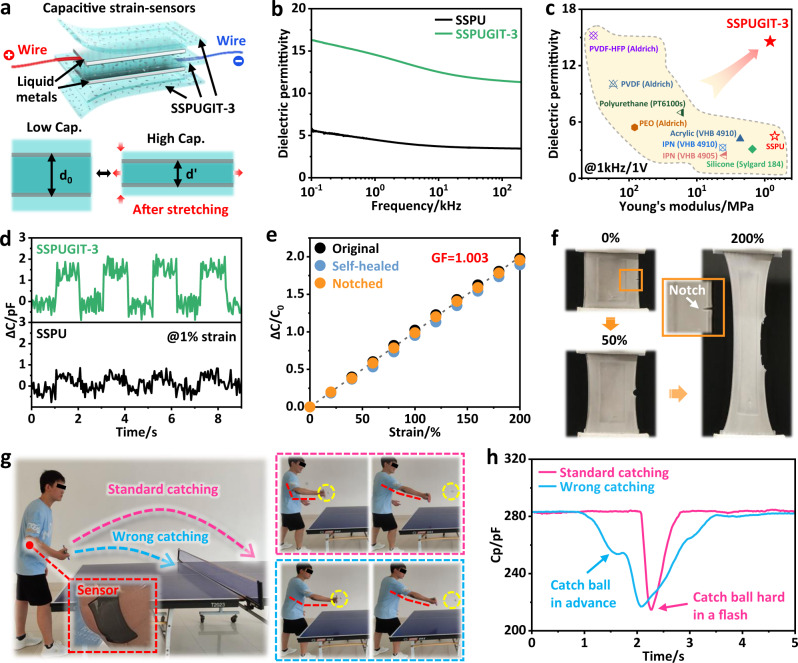
Application in self-healing capacitive strain-sensors. **a** Schematic illustration of a soft capacitive strain-sensor based on SSPUGIT-3 composite. **b** Dielectric permittivity of SSPU and SSPUGIT-3 as a function of frequency at 25 °C. **c** Comparison of the dielectric permittivity and Young’s modulus among commercial dielectric materials with SSPUGIT-3. **d** Cyclic tensile tests of SSPU- and SSPUGIT-3-based sensors at 1% strain with a fixed time interval of 1 s. **e** Relative capacitance change of original, self-healed and notched SSPUGIT-3-based capacitive strain-sensors as a function of tensile strain; GF reparents the gauge factor of sensors. **f** Optical images of a notched SSPUGIT-3-based capacitive strain-sensor upon stretching to 200%. **g** Optical images illustrating the standard and wrong table tennis catching motions. **h** Relative capacitance response of the standard and wrong table tennis catching motions.

Soft capacitive strain-sensors are crucial in emerging domains including stretchable electronics, soft robotics and biocompatible human-machine interfaces^61^. Such a capacitive strain-sensor usually consists of a soft dielectric layer sandwiched by conductive and packaging layers (Fig. 5a; Supplementary Fig. 34). Traditionally, stretching the sensor causes the top and bottom electrodes closer together, and thus increasing capacitance (C) according to a universal formula of *C* = *ε*_*0*_*ε*_*r*_*S*/4π*kd*, where d is the distance between electrodes, *ε*_*r*_ is the dielectric permittivity (*κ*) of dielectric layer (Fig. 5a)^62^. Therefore, capacitive sensitivity to a given strain of soft sensors is maximized by using high-κ materials^63^; however, most soft materials used today like silicones, rubbers, or polyurethanes possess relatively low-κ values. A typical sample is the SSPU, the *κ* value of which is only in a small range of 3.47–5.64 across the whole frequency range (Fig. 5b). Previously, various rigid fillers including metallic, ceramic, and carbon-based particles had been incorporated into soft polymer matrix to greatly enhance their dielectric properties, but this is always at the cost of intrinsic softness and compliance^64^. In essence, such modified materials generally show a characteristic of high rigidity/modulus and high-κ value, which is obviously incompatible with the high compliance required by soft and stretchable capacitive sensors. Intriguingly, our bioinspired SSPUGIT-3 composite was not only soft yet tough (Fig. 2), but also integrated high-*κ* value between 11.36 and 16.37, which was 3.27- to 2.92-fold higher than that of SSPU. Such a high-*κ* value arose from the excellent electrical polarizability of Galinstan droplets, which contributed to an amplified electrical displacement (*D*) upon the SSPUGIT-3 composite was subjected to an externally applied electrical field (*E*)^65^. According to the formula of *D* = *εE*, a larger *D* value determined a higher *κ* value. Meanwhile, dielectric dissipation of SSPUGIT-3 was as low as the unfilled SSPU (Supplementary Fig. 35), well within the threshold for dielectric functionality^66^. As a whole, our SSPUGIT-3 offered a unique combination of low modulus and high dielectric permittivity, far superior to many commercial dielectric materials (Fig. 5c; Supplementary Table 4). Of particular note was that SSPUGIT-3 was always electrically insulating even elongated to a large strain of 500% (Supplementary Fig. 36). All these prove its potential as a robust dielectric material tailored for soft capacitive strain-sensors (Supplementary Fig. 34).

The assembled capacitive strain-sensor based on SSPUGIT-3 dielectronic layer could quantitatively measure the external deformation to a high strain of 200% (Supplementary Fig. 37), which is due to the robust and stable interfaces between different layers. We observed that the loaded capacitance increased linearly along with stretching, matching well with the formula of *C* = *C*_*0*_*ΔL*/*L*_*0*_ (*L* is the length of capacitive sensor) to achieve a gauge factor (GF) of 1.003 (Supplementary Fig. 36). For comparison, we also fabricate a control capacitive strain-sensor by using SSPU as dielectric and packaging layers (Supplementary Fig. 38). As expected, the capacitive sensitivity at 1% strain of SSPUGIT-3-based sensor is much higher than that of SSPU-based sensor, together with a more distinct and regular capacitance curve (Fig. 5d). Meanwhile, the response time of SSPUGIT-3-based sensor is as low as 32 ms at 1% strain (Supplementary Fig. 39), which is comparable to previously reported soft capacitive strain-sensors^67^. Since the SSPUGIT-3 material showed fast and elastic deformation recovery at strain regions <60% (Supplementary Fig. 40), the integrated sensor could sensitively detect wrist-bending motions with ignorable hysteresis (Supplementary Fig. 41 and Movie S4). The whole sensor could also restore damages or cuts with a little sensitivity degradation because of the excellent self-healing performance of SSPUGIT-3 (Fig. 5e; Supplementary Fig. 42)^68^. Most remarkably, even if a mechanical notch (~2 mm) was made on the SSPUGIT-3-based sensors, it still continued to work due to its inherently ultra-high fracture toughness that restrained crack propagation under deformation (Fig. 5e, f; Supplementary Movie 5). In sharp contrast, the typical SSPU-based sensor ruptured at a strain of <135%, limiting its strain sensing capability with mechanical damages (Supplementary Fig. 43 and Movie 5).

Owing to its excellent strain sensitivity, remarkable crack resistance, ultra-high fracture toughness and fast self-healing capability, SSPUGIT-3-based capacitive strain-sensor was then employed to detect whether the motions or strokes met the demands in the table tennis training. Firstly, a soft capacitive strain-sensor was conformably mounted on the elbow of a ping-pong player (Fig. 5g). When he hit a ping-pong ball with a standard motion, i.e., keeping his elbows bent before hitting and fleetly applying external force upon hitting the ball, the capacitance line (pink) would exhibit a groove after a relative balance (Fig. 5h). However, if the ping-pong player made a wrong shot, i.e., his elbow was straightened in advance and thus hitting the ball, the capacitance line (blue) would slowly generate a step and then display a groove (Fig. 5h). Accordingly, such standard capacitance curves could be used by ping-pong players to correct their wrong catching actions, demonstrating a great application potential of our soft yet tough self-healing SSPUGIT composite.

## Discussion

In this study, we report, to the best of our knowledge, the first example of bioinspired materials to mimic the soft yet tough properties, crack-resistant ability and self-healing performance of human vascular smooth muscle tissues. This unique vascular smooth muscle-inspired material was readily achieved via a hierarchical structure design, that is, engineering core-shell structured spindle Galinstan micro-droplets into soft self-healing polyurea matrix through molecularly interfacial metal-coordinated assembly. The resultant SSPUGIT composite showed extraordinary enhancements of crack-resistant strain (from 121.66 ± 21.24% to 1541.75 ± 61.32%) and fracture toughness (from 3.18 ± 0.51 kJ m^−2^ to 111.16 ± 8.76 kJ m^−2^), which is 12.2 and 34.9 times higher than those of initial SSPU, respectively, and retained the intrinsic softness with an inappreciable increase in Young’s modulus (0.83 ± 0.05 MPa). Such a soft toughening mechanism is due to the multiscale cooperation for tremendous mechanical dissipation, in which stress concentration at crack tip was first alleviated through the breakage of Ga_2_O_3_ shell together with the rupture of interfacial coordination interactions in micro- and molecular scales, followed by complete elimination of primary crack that could adaptively moving along with highly elongated liquid Galinstan at mesoscale. Significantly, the SSPUGIT composite also possesses remotely controlled yet highly accurate NIR-induced fast self-healing and superior dielectric properties through the functionality of the vascular smooth muscle-like Galinstan microstructure. This extraordinary combination of properties allows SSPUGIT composite have a more central role in emerging stretchable electronics applications, where the devices’ robustness highly depends on the crack resistance, fracture toughness and healing performance of the soft materials.

## Methods

### Materials

Bis(3-aminopropyl)-terminated poly(dimethylsiloxane) (H_2_N-PDMS − NH_2_, M_n_ = 3000) was purchased from Gelest. Trimethyl hexamethylene diisocyanate (THI, AR, 97%) and 3,3′-dimethyl-4,4′-biphenylene diisocyanate (DBI, AR, 98%) were supplied by Aladdin Reagent Limited Corporation, China. Gallium–indium–tin eutectic alloys (Galinstan, GTS-1) were supplied by Zhenjiang Asian Electronic Technology Co., Ltd. in China. Chloroform (CHCl_3_, AR, 99%) was dried with the molecular sieves for 48 h before use.

### Synthesis of SSPU

4.0 g of H_2_N-PDMS − NH_2_ (M_n_ = 3000, 1 eq.) was dissolved in 15 mL anhydrous CHCl_3_ in a 50 mL two-necked round-bottomed flack equipped with magnetic stirrer under nitrogen atmosphere at room temperature. After stirring for 5 min, a mixture of THI (0.1682 g, 0.6 eq.) and DBI (0.1409 g, 0.4 eq.) pre-dissolving in 15 mL anhydrous CHCl_3_ was injected dropwise into the above H_2_N − PDMS − NH_2_ solution. Subsequently, the resulting solution was stirred for 2 h while the temperature was kept at 60 °C. Then, the obtained product was poured into a Teflon mold and dried at room temperature for 12 h, followed by dried at 60 °C for another 24 h to remove the residual solvent. Finally, Clean and dry SSPU film were peeled off for further characterizations. The control sample was synthesized by the same procedure with H_2_N-PDMS − NH_2_ chains possessing high molecular weight (M_n_ = 5000–7000).

### Fabrication of SSPUGIT composites

4.0 g of prepared SSPU elastomer was dissolved with 15 ml CHCl_3_ in a teflon beaker. Then, a certain amount of Gallium–indium-tin eutectic alloys (Galinstan) was incorporated into the SSPU solution for continuously mechanical stirring of 1 h at a high speed of 500 rpm (the mass ratio of Galinstan to SSPU is 1:1, 2:1, 3:1 and 4:1), aiming to reduce and stabilize the size of the Galinstan liquid fillers. Subsequently, this mixture was poured into teflon molds and dry at room temperature for 24 h to totally evaporate the chloroform. Finally, the obtained composite films were peeled off and then stored in a desiccator for future experiments.

### Fabrication of SSPUGIT-3-C composite

A SSPUGIT-3-C control sample with 300 wt% Galinstan was prepared to demonstrate the particularity of vascular smooth muscle-inspired structure for enhanced crack-resistant strain and fracture toughness. The specific preparation process of SSPUGIT-3-C is as follows. Firstly, a prefabricated monolithic SSPUGIT-3 sample was cut into pieces, and then suffer from extrusion through a miniature twin screw extruder. Afterwards, the obtained mixture was collected and transferred to a mold to undergo hot pressing. Finally, a dense SSPUGIT-3-C control sample was fabricated, which shows a chaotic structure due to the shearing process during extrusion.

### Fabrication of self-healing capacitive strain-sensors

Typically, the capacitive strain-sensor was consisted of a dielectric layer between the top and bottom conductive and packaging layers (Fig. 5a). Firstly, SSPUGIT-3 was totally dissolved, and then poured into a rectangular Teflon mold equipped with a rectangular protrusion in its middle. After drying, a unique SSPUGIT-3 film with a rectangular shallow groove was peeled off for using as electrode encapsulation material. Subsequently, little liquid metal alloy was bladed to fill the groove as the conductive layer. Lastly, an ideal capacitive strain-sensor with a total thickness of ~1.2 mm was fabricated by sandwiching the other smooth SSPUGIT-3 film (that is used as dielectric layer) with two encapsulated electrodes. The control capacitive strain-sensor using SSPU as dielectric and packaging layers was fabricated at the same procedures.

### General characterization

^1^H NMR spectra were recorded on Bruker AVANCE III 500 MHz spectrometer at room temperature with tetramethylsilane (TMS) as an internal reference. ATR-FTIR spectra were recorded on a Bruker Tensor II spectrometer that equipped with a Specac Golden Gate ATR heating/cooling cell in the range of 4000–600 cm^−1^. UV–vis transmittance spectra were recorded by a Thermo Scientific E220 UV-vis spectrophotometer. SAXS analysis were operated on the Bruker AXS NanoSTAR instrument equipped with a microfocus X-ray source, operating at *λ* = 0.1541 nm. DSC measurement was performed on a TA DSC−25 differential scanning calorimeter at the heating rate of 20 °C min^−1^. SEM measurements and the corresponding mappings were performed with a JEOL 7800 F field emission electron microscope under an acceleration voltage of 2 kV. Optical microscopy images were recorded using a Jiangnan MV3000 optical microscope. Infrared thermal images and the temperature variation under 808 nm NIR light irradiation were recorded by a A615 Infrared Thermal Imager. The capacitance and dissipation factor of the assembled strain-sensors were measured with a precision Agilent 4294 A impedance analyzer, then the corresponding dielectric constants were inversely calculated.

### Mechanical characterization

Tensile tests were conducted on a Shimadzu AGS-X tester at 25 °C with a strain rate of 100 mm min^−1^. Five specimens with a tensile size of 10 mm gauge length × 6 mm width × 0.3–0.5 mm thickness were tested, and their average value was given. Indeed, Young’s modulus was calculated from the fitted slope of the initial stress-strain curves in tensile test. For the cyclic tensile test, both loading and unloading process were performed at a strain rate of 100 mm min^−1^ at 25 °C. Pure shear tests were performed to evaluate the fracture toughness and crack resistant strain. A notch of 20 mm in length was first made in a rectangular specimen (60 mm width × 0.3–0.5 mm thickness). Then, the intact and notched specimens were subjected to tension with a gauge length of 10 mm. The fracture toughness is calculated as *Г* = *W*_c_*H, where *W*_*c*_ is the strain energy density by the applied force to the unnotched sample until critical strain (that is the crack resistant/failure strain of the notched sample), and *H* is the initial gauge length between the two clamps.

DMA was conducted on a TA Q800 instrument in the film tension geometry. Dimensions of the specimens for DMA measurements were determined by a standard Vernier calliper, and meanwhile, the length of the specimens between the tensile clamps was determined by the DMA instruments itself. The temperature sweep experiments were conducted under tension condition with a frequency of 1 Hz. The scanning temperature ranged from −50–100 °C at a heating rate of 5 °C min^–1^. For stress relaxation experiment, all samples were quickly stretched to 200% strain and then set at this strain for relaxation for 20 min. Subsequently, the applied stress on these samples were gradually relaxed in different degree. Bulk rheological measurements were performed on TA DHR-1 Rheometer with a 20 mm parallel steel plate. Frequency sweeps were performed at a rotational strain amplitude of 0.1% by varying the frequency between 0.1 and 100 rad s^−1^ in a temperature range of 25–95 °C.

### Self-healing testing

For self-healing tests of mechanical properties for SSPUGIT, each spline was cut into two completely separate pieces with a razor blade. Subsequently, the cut faces were gently contacted together without any press, and then irradiated by NIR laser at 1.99 W cm^−2^ NIR laser within 1 min to enable restoration. Afterwards, the healed splines were subjected to tensile test at 25 °C with a tensile rate of 100 mm min^−1^. The healing efficiency, η, was calculated from the ratio of the integral areas under stress-strain curves of healed splines to that of the virgin one under stress-strain curves. The measurements were performed using more than three splines for each healing time to achieve the average value. The self-healing process of SSPU was accomplished at ambient temperature or high temperature for various healing times, which did not response to NIR light. Meanwhile, the self-healing efficiency of SSPU was evaluated in the same way to that of SSPUGIT.

Towards self-healing test of electrical properties for the assembled capacitive strain-sensors, the self-healing process was performed as described above under the monitoring of impedance analyzer. Specifically, the cut faces were brought back into contact and then irradiated by NIR light at 1.99 W cm^−2^ to achieve healing. Then, the self-healed sample was taken to measure the variations of capacitance as a function of tensile strain.

### Consent statement

The authors affirm that human research participants provided informed consent for publication of the images in Fig. 5g and Supplementary Fig. 41.

## Supplementary information

Supplementary Information

Description of Additional Supplementary Files

Supplementary Movie 1

Supplementary Movie 2

Supplementary Movie 3

Supplementary Movie 4

Supplementary Movie 5

## Data Availability

All relevant data supporting the results of this study are available within the article and its supplementary information files. Further data are available from the corresponding authors upon request.
