# Supplementary material for: Vascular smooth muscle-inspired architecture enables soft yet tough self-healing materials for durable capacitive strain-sensor
Source: Nat Commun. 2023 Jan 10;14:130. doi: 10.1038/s41467-023-35810-y (PMC9829674; doi:10.1038/s41467-023-35810-y)
Supplement: Supplementary file 1 — Supplementary Information [file 41467_2023_35810_MOESM1_ESM.pdf]

Supplementary Information

Vascular smooth muscle-inspired architecture enables soft yet tough self-healing materials for durable capacitive strain-sensor

FuYao Sun[1,2], LongFei Liu[1], Tong Liu[1,3], XueBin Wang[1,4], Qi Qi[5], ZuSheng Hang[3], Kai Chen[5], JianHua Xu[1,2,4*], JiaJun Fu[1,4*]

1. School of Chemistry and Chemical Engineering, Nanjing University of Science and Technology, Nanjing 210094, China

2. Jiangsu Co-Innovation Center of Efficient Processing and Utilization of Forest Resources, College of Chemical Engineering, Nanjing Forestry University, Nanjing 210037, China

3. School of Materials Science and Engineering, Nanjing Institute of Technology, Nanjing 211167, China

4. State Key Laboratory of Coordination Chemistry, Nanjing University, Nanjing 210093, China

5. School of Science, Nanjing University of Science and Technology, Nanjing 210094, China

*Corresponding author. E-mail: xujianhua931118@163.com; fujiajun668@gmail.com

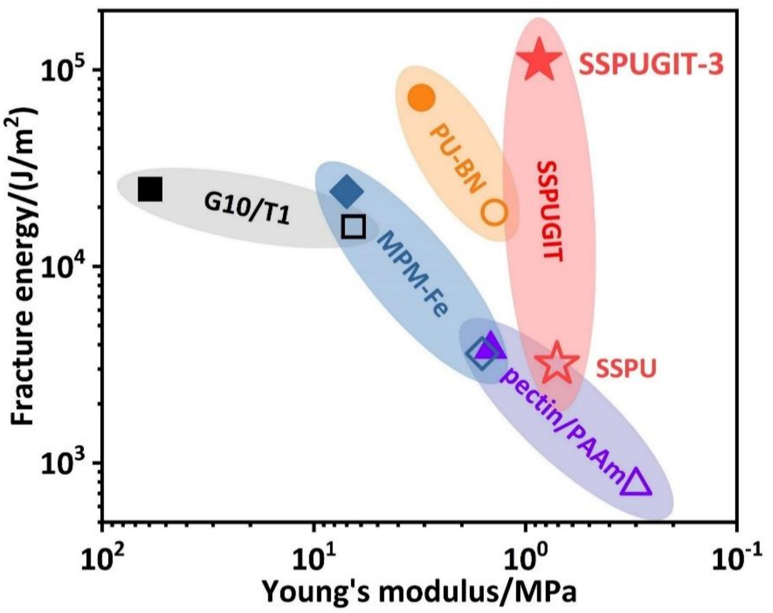

Supplementary Figure 1. Comparison of the fracture energy versus Young's modulus among other toughened materials with SSPUGIT. As shown, various reported methods aiming to improve the fracture energy/toughness would lead to the increase of Young's modulus, i. e., sacrificing materials' intrinsic softness (Supplementary Table 5)[1-4]. In sharp contrast, our bioinspired strategy retained the intrinsic softness of materials to the greatest extent while extremely toughening them.

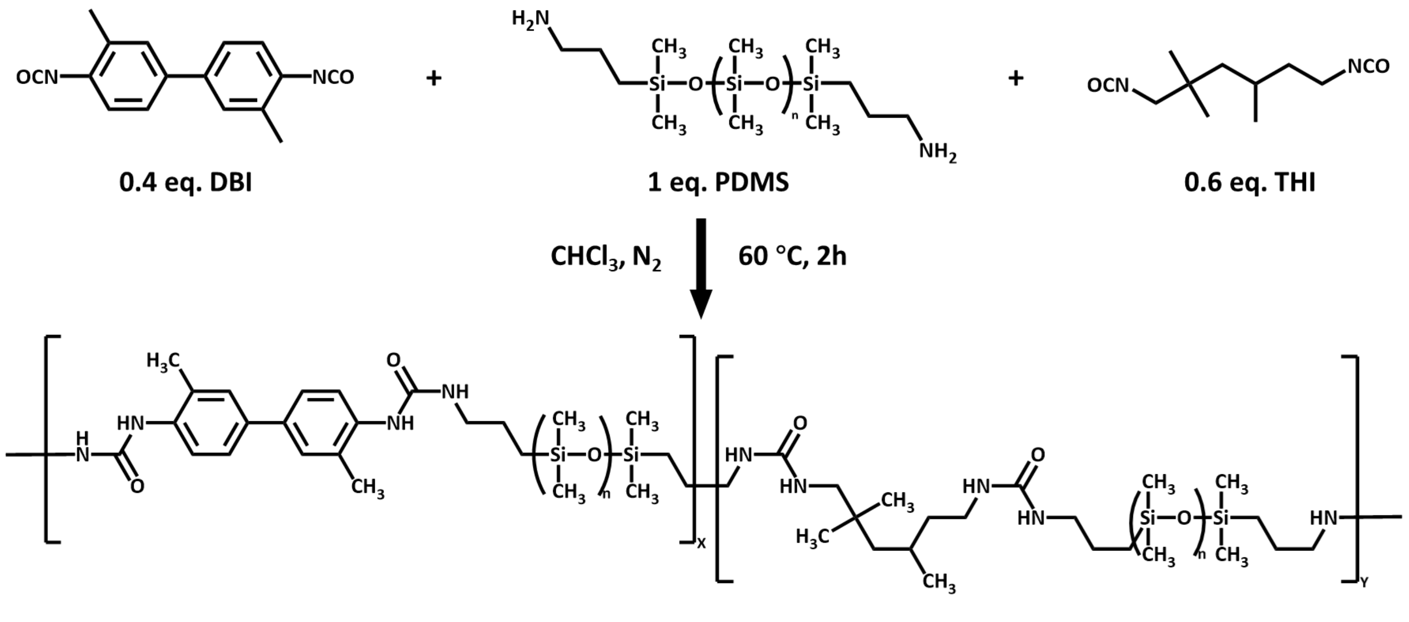

Supplementary Figure 2. Schematic illustration of the synthetic procedure of SSPU. The SSPU elastomer was synthesized through a one-pot polycondensation reaction between bis(3-aminopropyl)-terminated poly(dimethylsiloxane) (PDMS, M_n=3000) and a mixture of 3,3'-dimethyl-4,4'-biphenylene diisocyanate (DBI) and Trimethyl hexamethylene diisocyanate (THI).

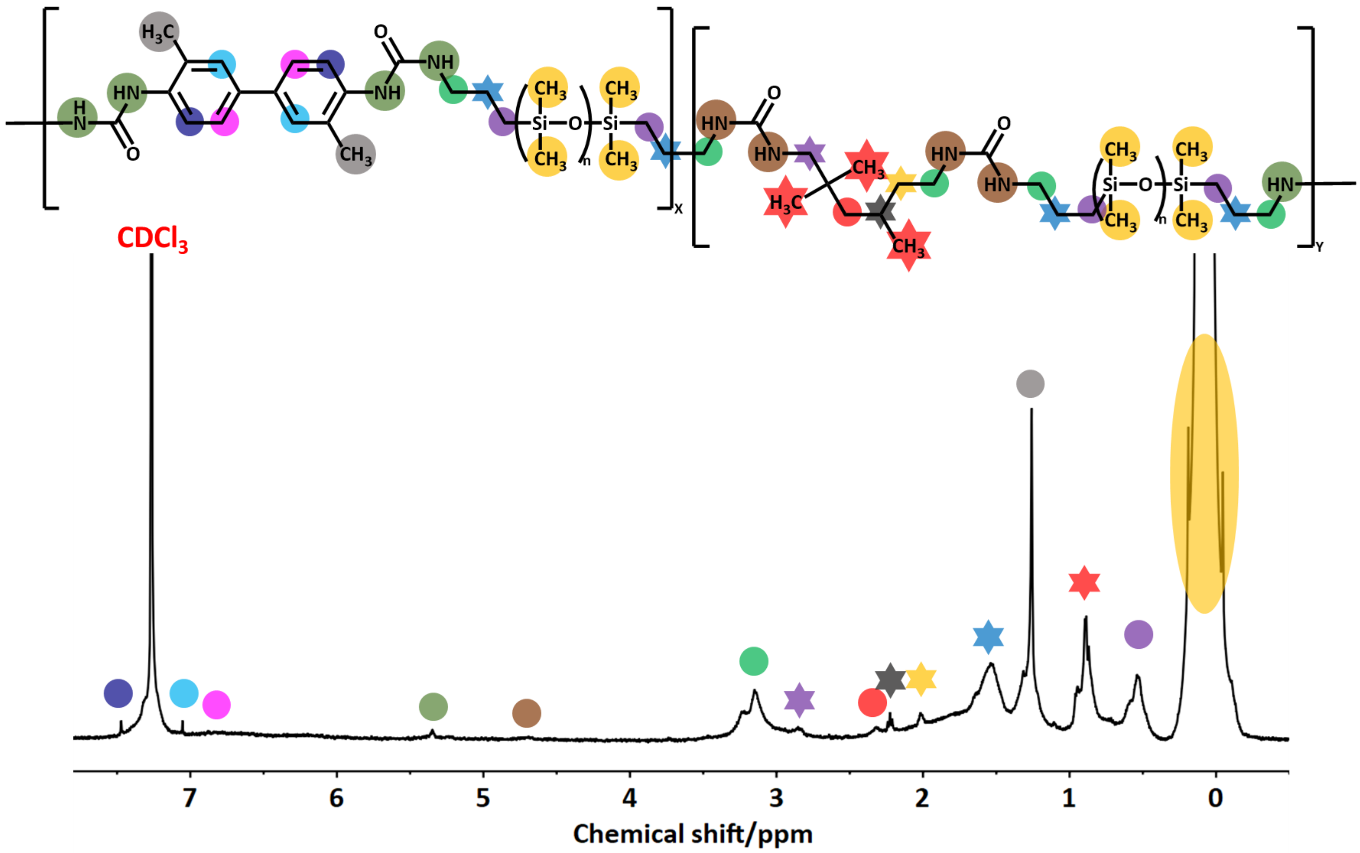

Supplementary Figure 3. ^{1}H NMR spectra of SSPU (500 MHz, $CDCl_3$, 298 K). 0.06 ppm (H_3C-Si-CH_3), 0.53-1.53-3.15 ppm (-Si-CH_2-CH_2-CH_2-), 4.69-3.16-2.01 ppm (NH-CH_2-CH_2-), 2.23-2.32 ppm (-CH-CH_2-C-), 0.9 ppm (-C-CH_3), 2.85 ppm (-C-CH_2-), 5.34 ppm (Ph-NH-), 1.27 ppm (Ph-CH_3), 6.79-7.05-7.47 ppm (Ph-H).

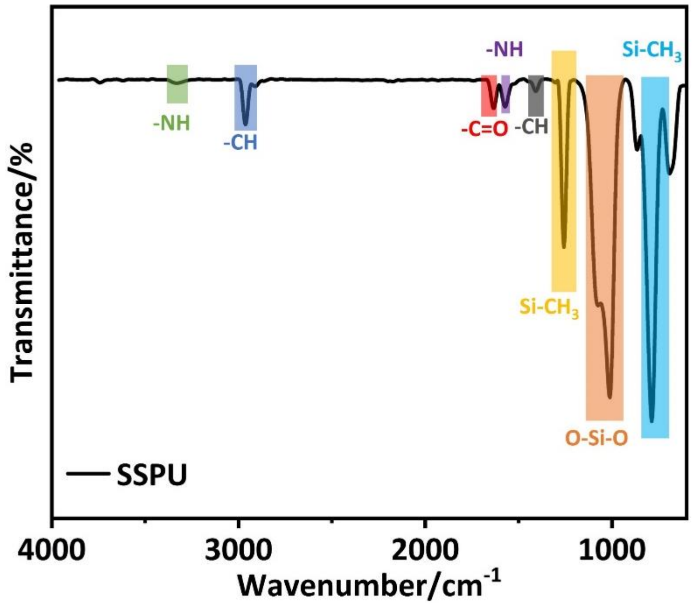

Supplementary Figure 4. FT-IR spectra of SSPU. As shown, FT-IR spectra displayed three characteristic peaks centered at 3332 cm^{-1}, 1634 cm^{-1} and 1572 cm^{-1}, which were assigned to the stretching vibration of -NH from amino group, stretching vibration of -C=O from urea groups and the flexural vibrations of -NH from amino group, respectively, demonstrating the successful synthesis of SSPU via the nucleophilic addition reaction between -NCO and -NH_2 motifs.

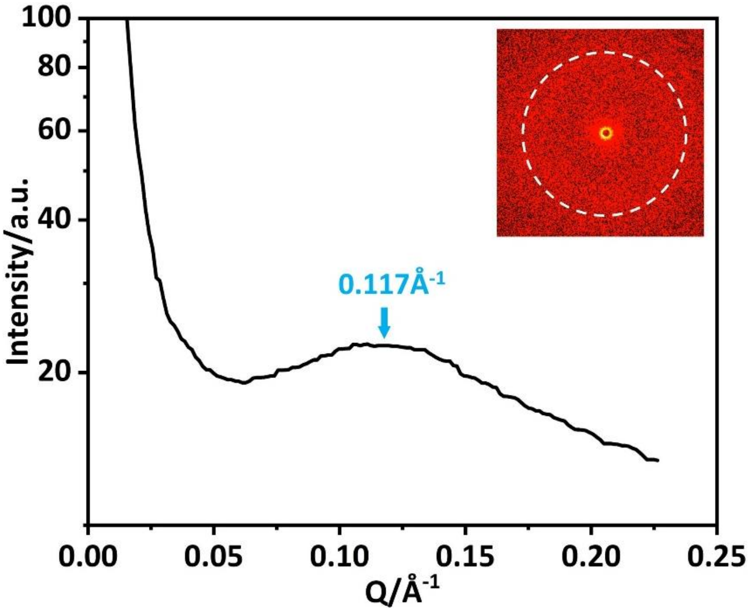

Supplementary Figure 5. SAXS spectra of SSPU, inserting image showing the distinct two-dimension grazing-incidence small-angle X-ray scattering (2D-GISAXS) pattern of the corresponding sample. As depicted, SSPU exhibited isotropic scattering behavior with a characteristic peak at q~1.17 nm^{-1}, demonstrating the formation of microphase separated structure[5]. However, the peak intensity (that is electron density contrast between hard and soft domains) was relatively weak, suggesting a loose and dynamic microstructure derived from self-sorting of DBU and THU units[5].

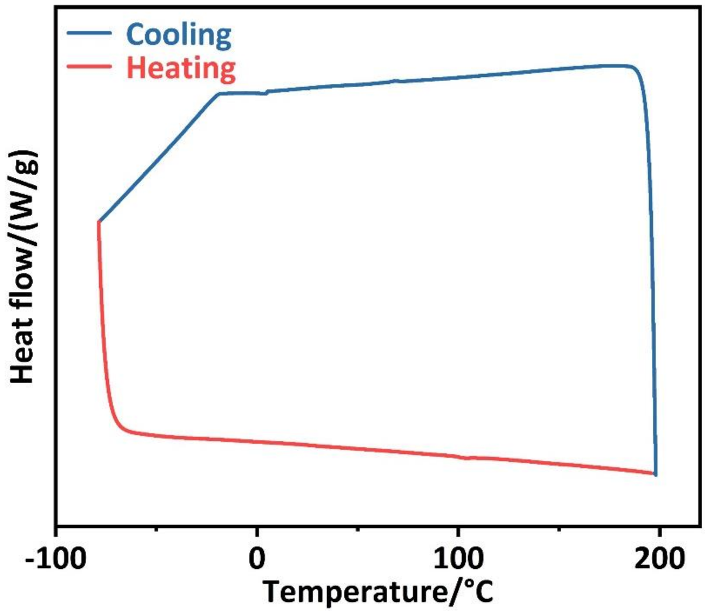

Supplementary Figure 6. DSC curves of SSPU. As shown, there was no obvious melting endothermic peak and crystallization exothermic peak during the heating and cooling process, respectively, demonstrating the amorphous microstructure of SSPU.

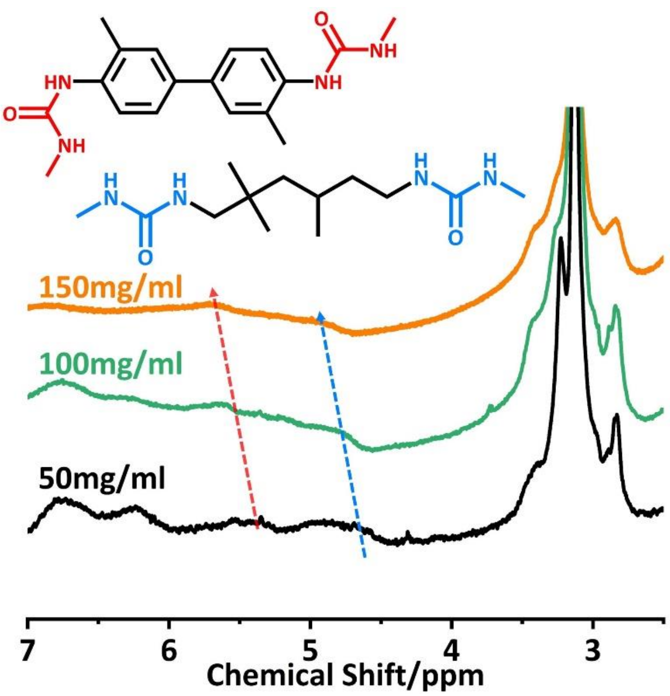

Supplementary Figure 7. Concentration-dependent ^{1}H NMR spectra of SSPU. As shown, two N-H protons derived from DBU and THU, respectively, gradually moved towards low fields with increasing concentration, demonstrating the formation of multiple hydrogen bonds in SSPU network[6].

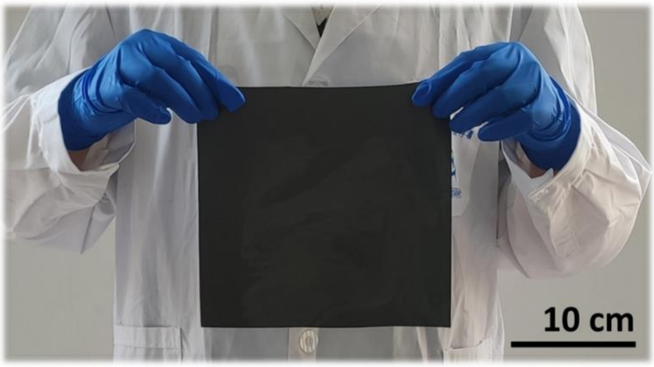

Supplementary Figure 8. Photograph of the large scale fabricated SSPUGIT-3 composite.

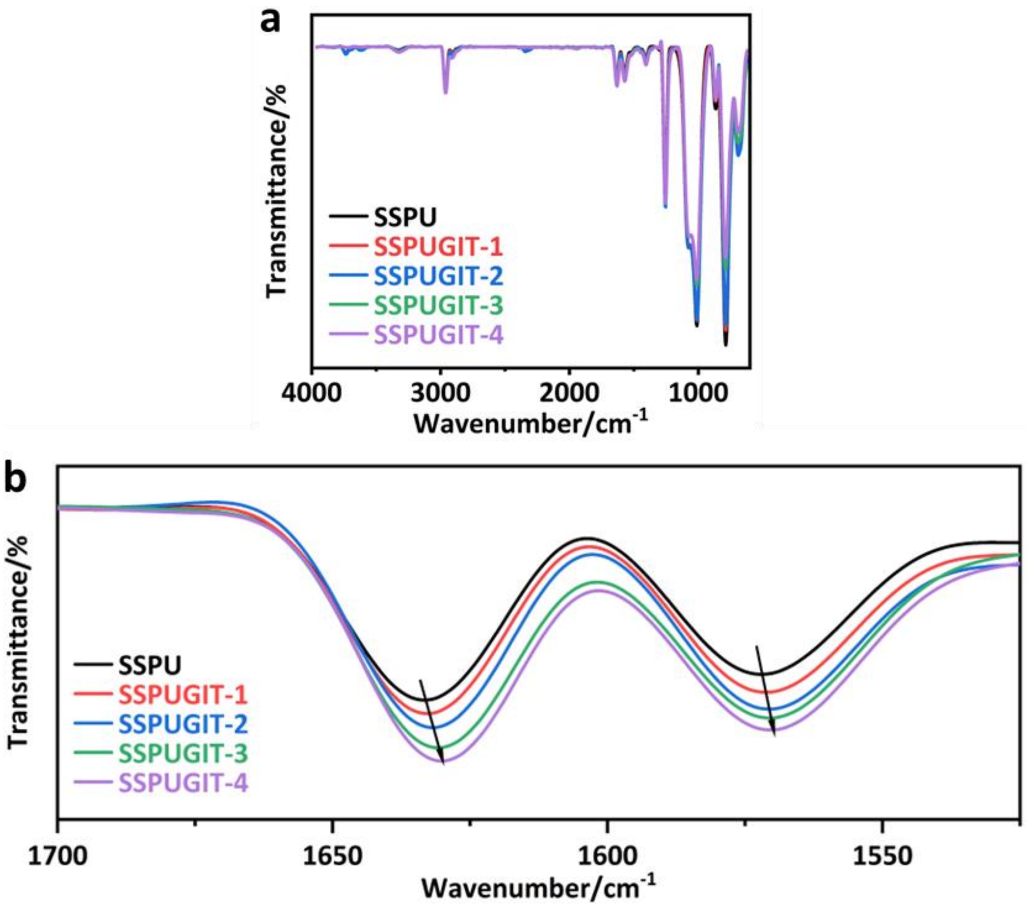

Supplementary Figure 9. FT-IR spectra of SSPUGIT composite; (a) full spectra from 4000-600 cm^{-1}; (b) in the region of 1700-1525 cm^{-1}. As shown, the peak centered at 1634 cm^{-1} (that is assigned to -C=O motifs) and 1572 cm^{-1} (that is assigned to -N-H motifs) of SSPU gradually shifted to the low wavenumber regions with the increase of Galinstan fillers, indicating the formation coordination interactions between SSPU and Ga_2O_3 shell[7]. These results also confirmed the successful fabrication of SSPUGIT composite.

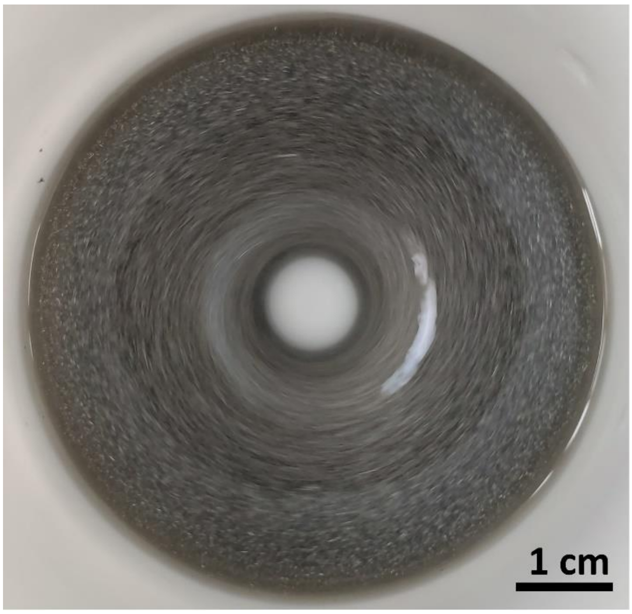

Supplementary Figure 10. Photograph of stirring high viscosity polymer solution and Gallium–indium-tin eutectic alloys during fabrication process of SSPUGIT. As shown, Galinstan droplets were gradually decreased in size to form uniformly spindle shape via a rotational shearing during continuous stirring.

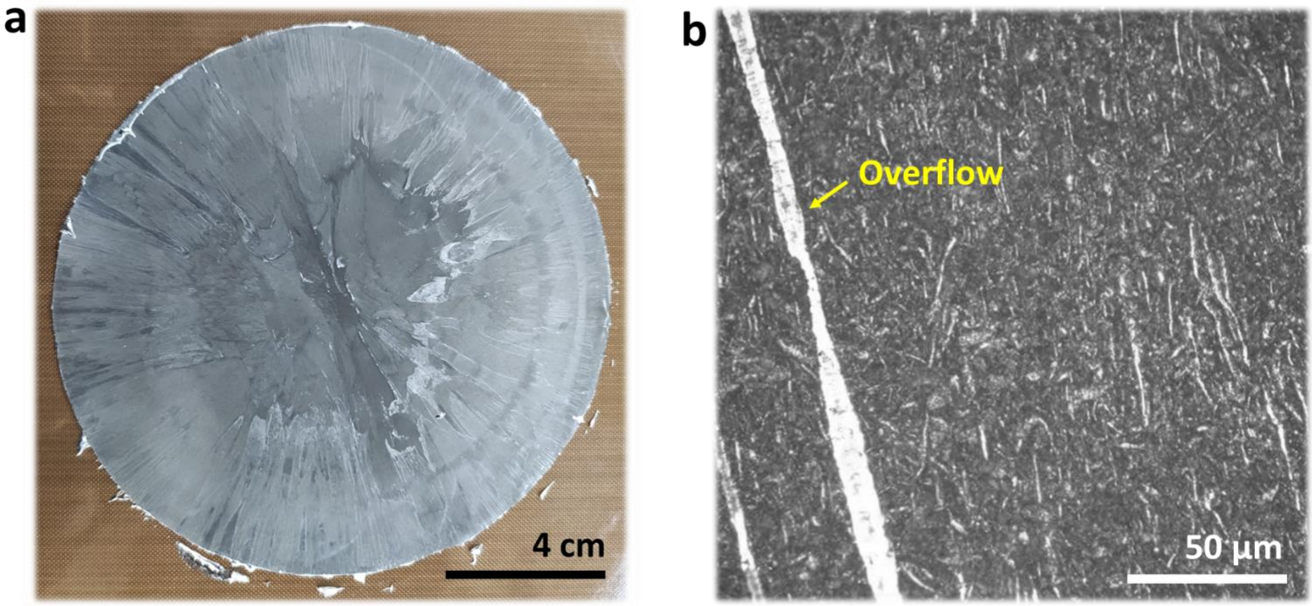

Supplementary Figure 11. (a) Photograph of SSPUGIT-C-3 control sample. (b) Optical microscopy image of SSPUGIT-C-3. As shown in Supplementary Fig. 11a, the fabricated SSPUGIT-C-3 control sample was heterogeneous, where the Galinstan was partially escaping from SSPU matrix due to the extrusion combined with hot pressing method. These overflow features were further conformed by optical microscopy image (Supplementary Fig. 11b). As depicted, vast majority of Galinstan was distributed in SSPU in a messy way, which is totally different with the vascular smooth muscle-inspired structure of SSPUGIT-3 (Fig. 1e). Meanwhile, the size of the Galinstan droplets in SSPUGIT-3-C is much smaller than that of SSPUGIT-3, indicating that original micron-sized Galinstan droplets in SSPUGIT-3 were broken into more and smaller ones during the extrusion combined with hot pressing process, together with the formation of many rigid gallium oxide particles agglomerated in SSPUGIT-3-C matrix. High content of filler agglomeration would weaken the tensile stress and strain inevitably[8], leading to the poor mechanical behavior of SSPUGIT-3-C (Fig. 2a; Fig. 2c).

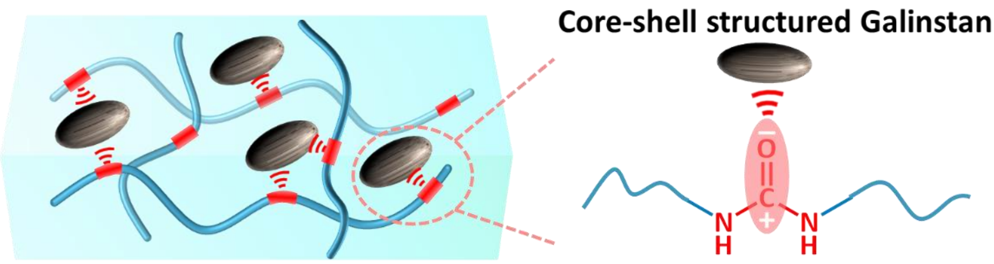

Supplementary Figure 12. Schematic illustration of the robust interfacial coordination interaction between Core-shell structured Galinstan and SSPU.

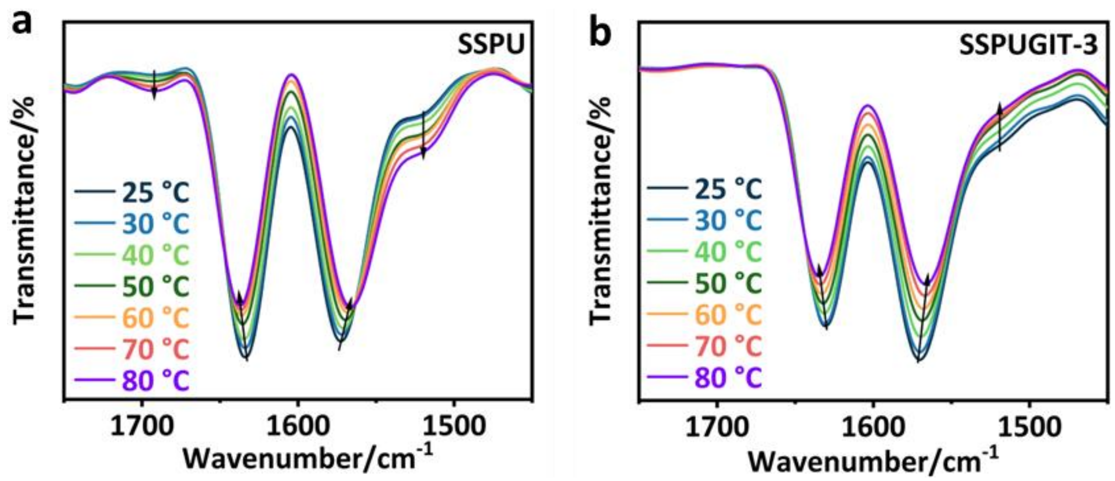

Supplementary Figure 13. Temperature-dependent FT-IR spectra of (a) SSPU and (b) SSPUGIT-3. For SSPU, when the temperature increases from 25 to 80 °C, the intensity of peaks at 1634 cm^{-1} (H-bonded -C=O motifs in urea) and 1572 cm^{-1} (H-bonded -N-H motifs in urea) gradually decreased, and the peaks at 1692 cm^{-1} (free -C=O motifs in urea) and 1519 cm^{-1} (free -N-H motifs in urea) gradually increased. These spectral features suggested the hydrogen bonding between different urea moieties gradually broken with the increase of temperature. However, as for SSPUGIT, the peak intensity of H-bonded -C=O motifs continued to decrease during the whole heating process, while the peak intensity of the free -C=O motifs is not changed at all. The phenomenon suggested that there were no free -C=O motifs generated in heating, probably due to the formation of robust coordination interaction between -C=O motifs and Ga_2O_3 shell[9].

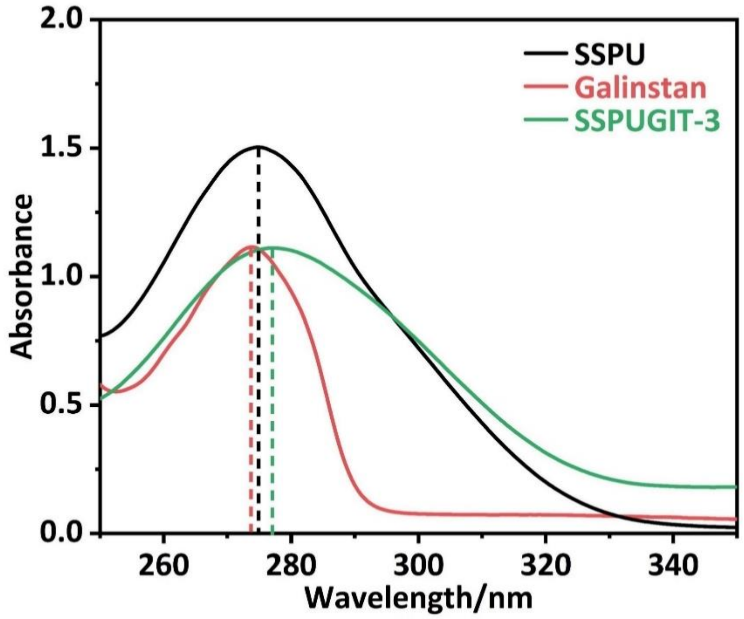

Supplementary Figure 14. UV–vis absorption spectra of SSPU, Galinstan and SSPUGIT-3 dissolved or dispersed in trichloromethane. As shown, the characteristic peak of SSPU dissolved in trichloromethane was centered at 274.81 nm, while the characteristic plasmon resonance for Galinstan particles dispersed in trichloromethane was centered at 273.62 nm. However, these peaks shifted to a higher wavelength of 276.90 nm for SSPUGIT-3, which is attributed to the coordination interaction between -C=O motifs and Ga_2O_3 shell[10].

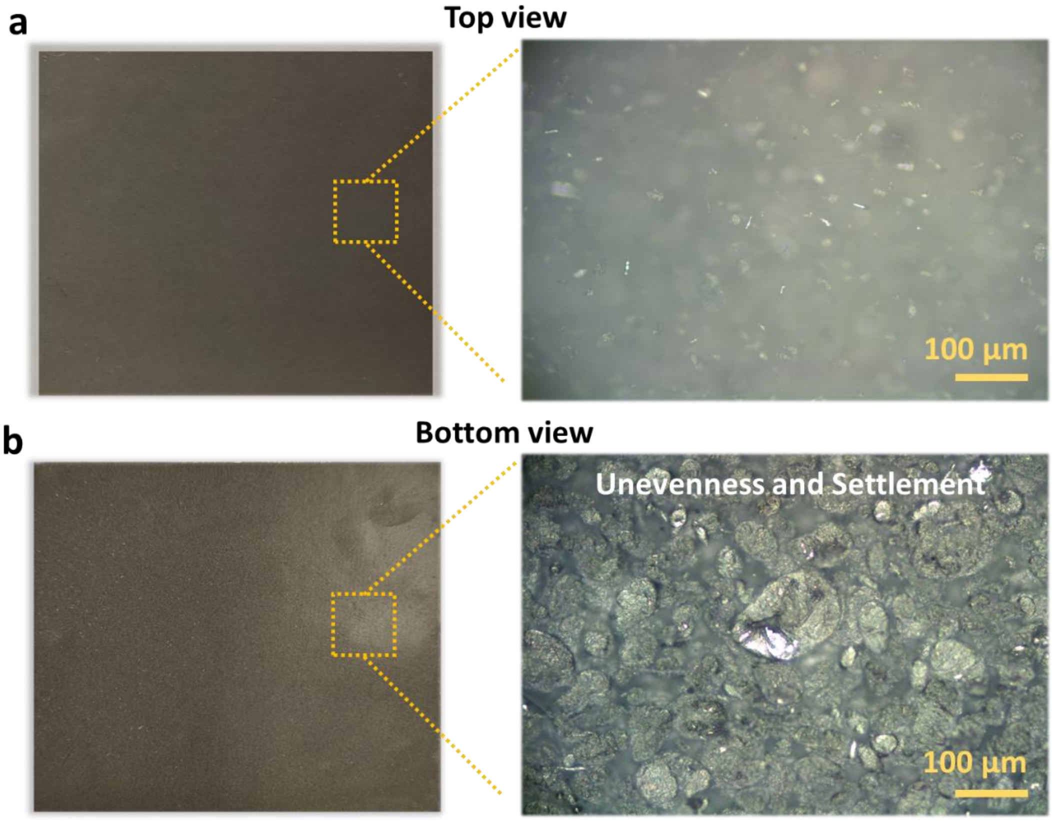

Supplementary Figure 15. (a) Top view and (b) bottom view of photographs and the corresponding optical microscopy images of the fabricated control composite, in which the SSPU matrix was synthesized by PDMS chains with a high molecular weight (M_n = 5000-7000 g mol^{-1}). As depicted in the photograph, the top view of the control composite looked uniform, but its bottom image exhibited obviously inhomogeneous argent particles. Meanwhile, as shown in optical microscopy images, Galinstan droplets were rarely visible in the top view, which settled and accumulated at the bottom of material to form macro-phase separation. Moreover, the size of the Galinstan droplets was heterogeneous, which may be due to the lack of sufficient interfacial interactions between SSPU chains and Galinstan to constrain agglomeration behavior. These phenomena indicated that the robust interfacial coordination interaction derived from -C=O motifs and gallium oxide shell was indispensable, which was a necessary prerequisite of successfully forming vascular smooth muscle-inspired structure to achieve soft yet tough self-healing materials.

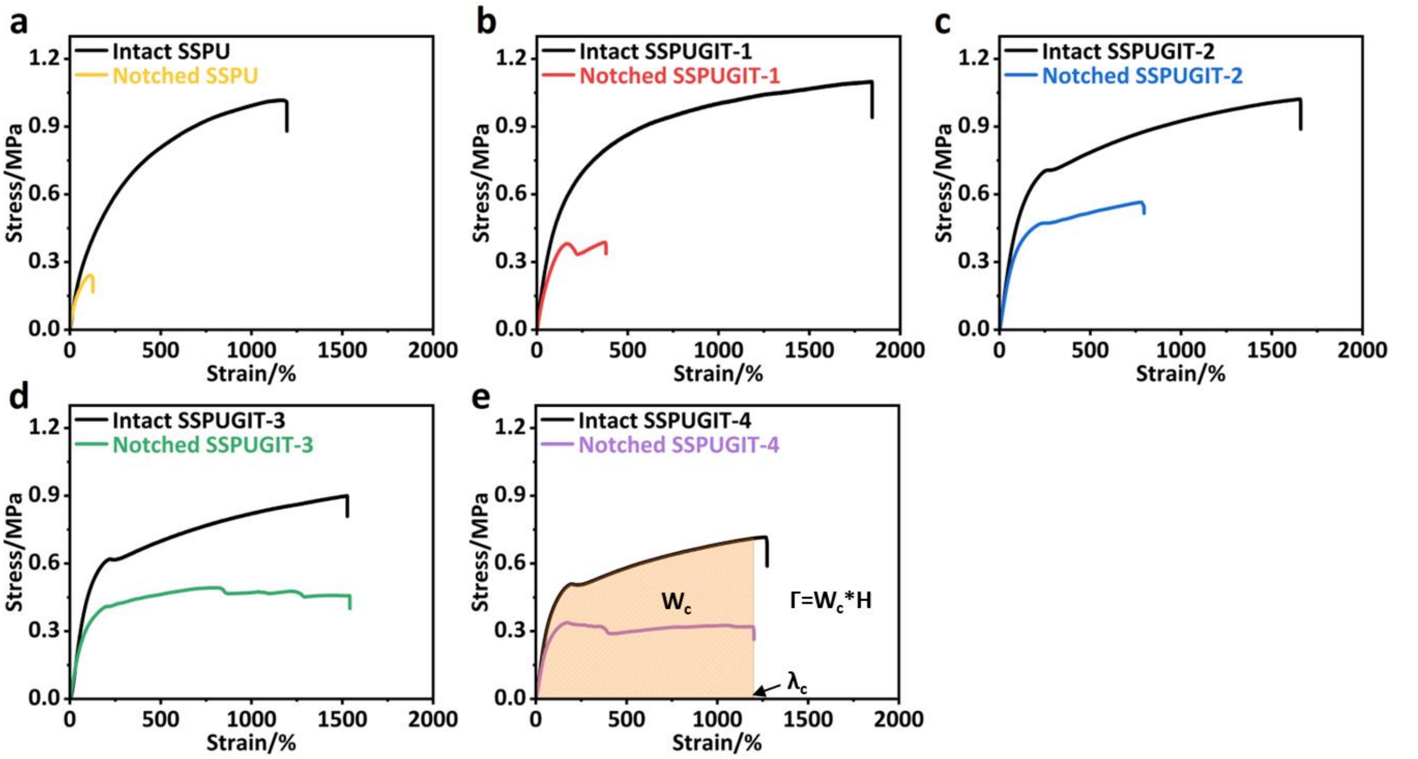

Supplementary Figure 16. Tensile stress–strain curves of the intact and notched (a) SSPU, (b) SSPUGIT-1, (c) SSPUGIT-2, (d) SSPUGIT-3 and (e) SSPUGIT-4 sample in pure-shear test. All the intact samples possessed a width of 60 mm, a gauge length of 10 mm, and a thickness of 0.3-0.5 mm; for notched samples, a 20 mm notch was made on the widthwise edge of the sample. As depicted, there was a critical value for the increase of crack resistant strain and the corresponding fracture energy, which suddenly dropped by increasing Galinstan content to 400 wt%. According to Rivlin-Thomas single-notch method[11], fracture energy (Γ) was proportional to strain energy density (W_c), i.e., $\Gamma \propto W_c$. However, the W_c value of SSPUGIT-4 was smaller than that of SSPUGIT-3 composite. By comparison, both SSPUGIT-3 and SSPUGIT-4 were highly crack-resistant that the notched sample possessed almost the same tensile strain as the intact sample. However, the tensile stress and tensile strain of the intact samples of SSPUGIT-4 is smaller than that of SSPUGIT-3 (black lines in Supplementary Fig. 16), which directly resulted in the decrease of W_c for SSPUGIT-4 composite. As shown in Supplementary Fig. 16, the tensile stress and tensile strain of the intact sample of SSPUGIT composite gradually decreased as Galinstan content increases. These reductions were in line with the classical linear elastic fracture mechanics (LEFM) theory that composites with much more filler droplets would break at lower stress (and hence lower strain) than composites with less droplets[12]. From another perspective, the droplets within composite matrix acted like the flaw. The reduced tensile strain also agreed with the theory than tensile strain markedly decreases when the flaw number increases[13]. For these reasons, the strain energy density (W_c) of SSPUGIT-4 decreased, and the corresponding fracture energy decreased as well.

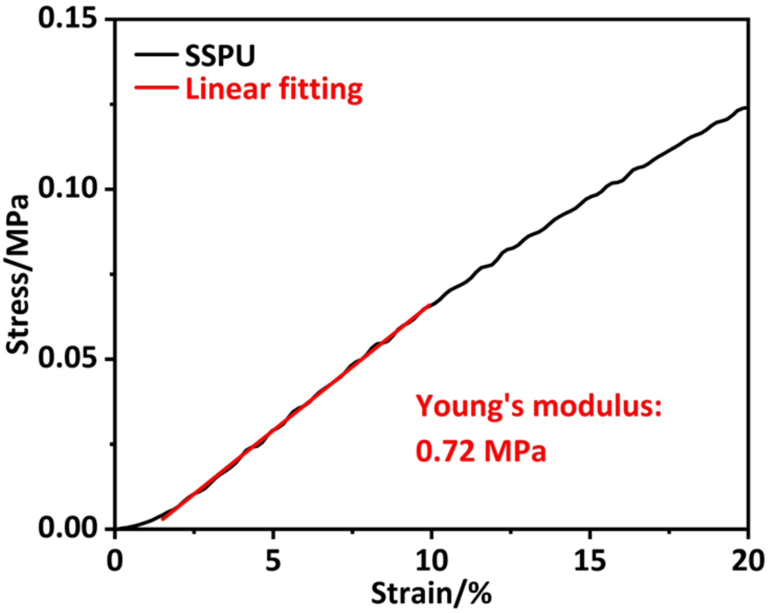

Supplementary Figure 17. The initial prats of the stress-strain curve of tensile test of SSPU. As shown, this curve was linearly fitted to achieve the slope, which corresponded to the Young's modulus of SSPU.

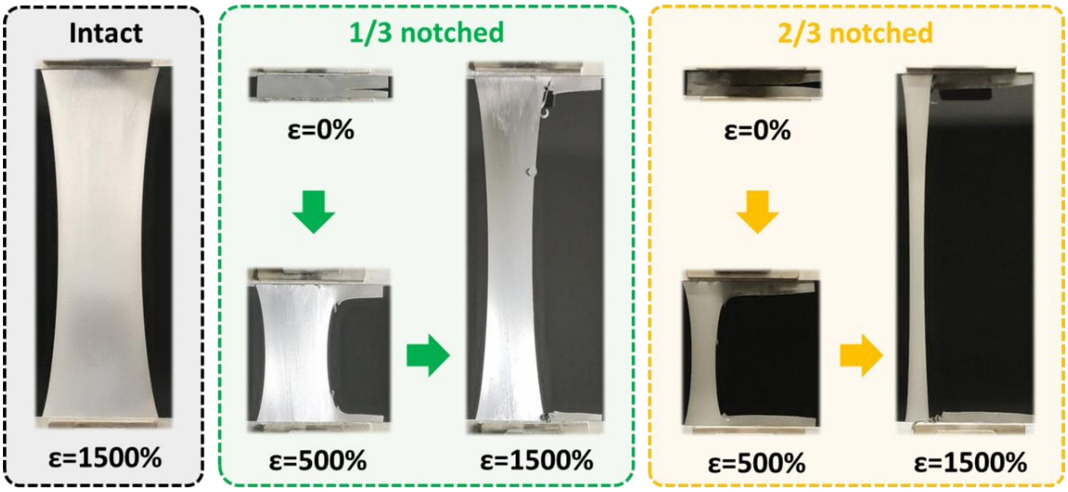

Supplementary Figure 18. Photographs of SSPUGIT-3 with different ratios of notch in tensile test. As shown, the notched sample could reach the same tensile strain of the intact sample, even if the proportion of notch to width was up to 2/3.

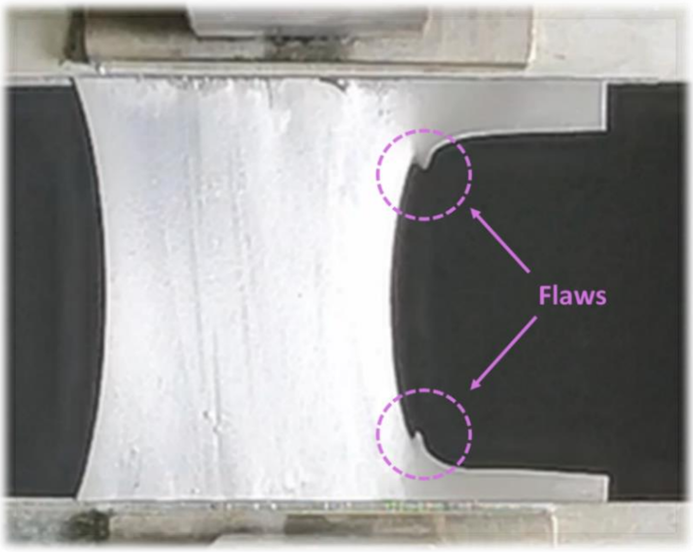

Supplementary Figure 19. Photograph of the generation of two flaws during stretching process for SSPUGIT-3 composite.

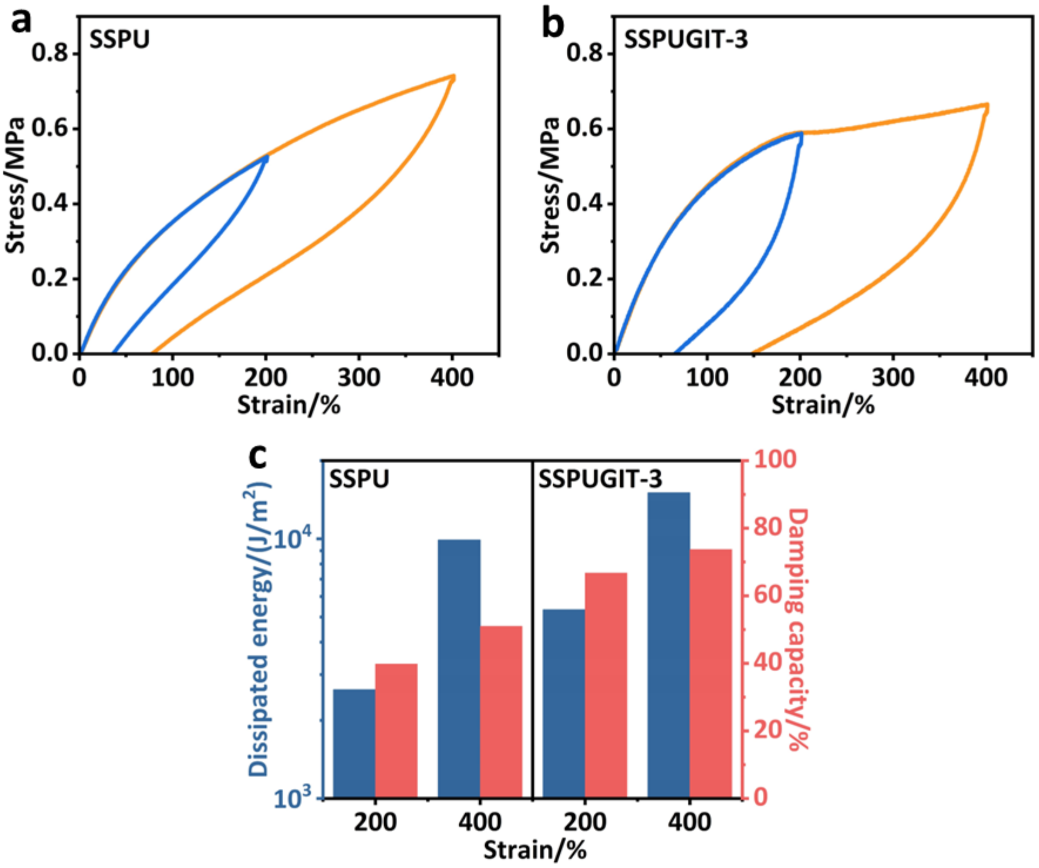

Supplementary Figure 20. Cyclic tensile properties of SSPU and SSPUGIT-3. (a) Cyclic tensile curves of (a) SSPU and (b) SSPUGIT-3 with the maximum strain of 200% and 400%. (c) Dissipated energy and damping capacity of SSPU and SSPUGIT-3 at different cyclic strains. The dissipated energy was defined as the energy dissipated during loading and unloading process, which could be calculated by integrating the area of cyclic tensile curves[11]. The damping capacity was defined as the ratio of energy dissipation to incoming energy[14]. As shown, SSPUGIT-3 dissipated more energy than SSPU at each strain (200%, 400%), which was attributed to the breakage of outer Ga_2O_3 shell and rupture of the interfacial coordination interactions between Ga_2O_3 and SSPU.

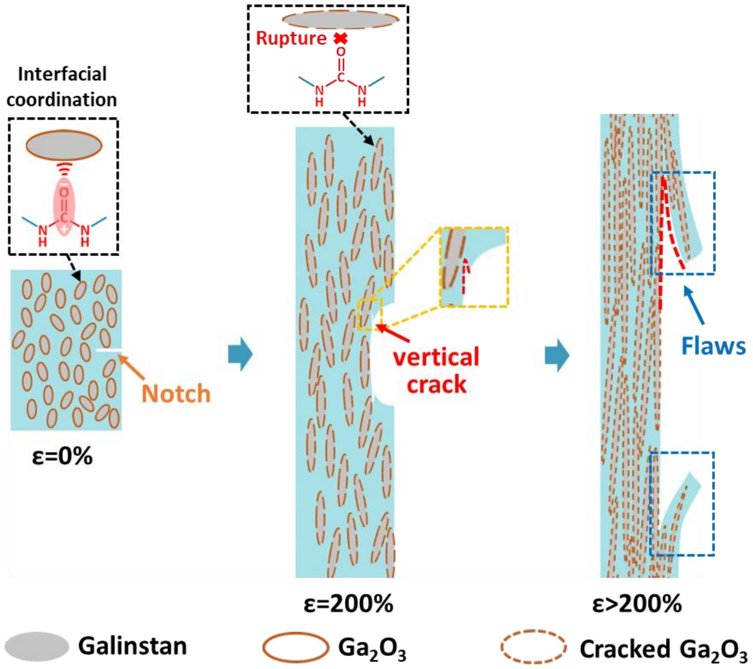

Supplementary Figure 21. Schematic illustration of deflection, branching and elimination crack for SSPUGIT composite. As shown, with the increase of tensile strain to ~200% strain, outer Ga_2O_3 shell of Galinstan particles was first broken and the interfacial coordination interactions between Ga_2O_3 and SSPU matrix was then destroyed accordingly. Subsequently, the inner liquid cores were released and further elongated into fiber-like ellipsoids as we continued to stretch (>200%).

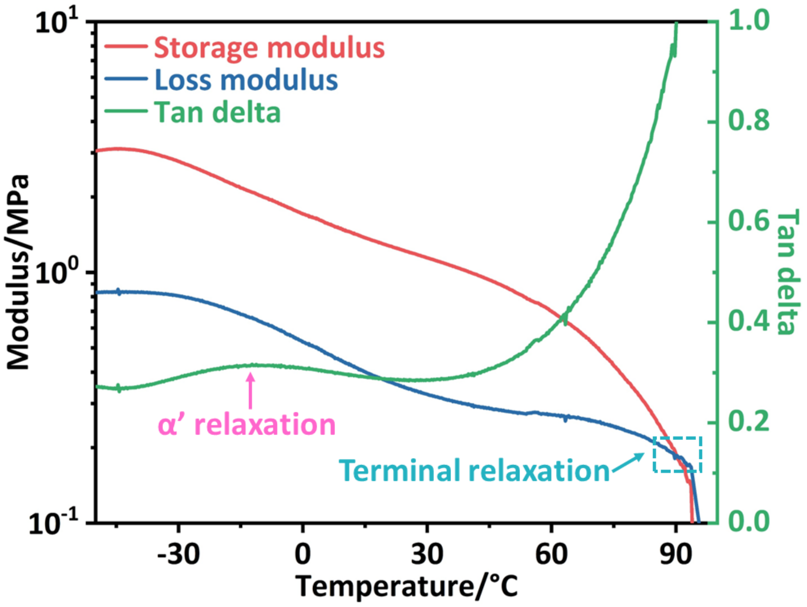

Supplementary Figure 22. Storage modulus, loss modulus and loss factor versus temperature for SSPU. As shown, there were two distinct relaxation process at -13.21 °C and 90.22 °C, which was assigned to the dynamics of hard domains (α' relaxation) and terminal relaxation of polymer chains (that is the crossover temperature between storage and loss modulus), respectively. According to loss factor curve (green line), the terminal relaxation started at room temperature, and was fully accompanied by an increase in tan δ to high temperature. This phenomenon was also reported by our group[15], manifesting that polymer chains had a tendency to flaw at room temperature, thus leading to the good room-temperature self-healing kinetics.

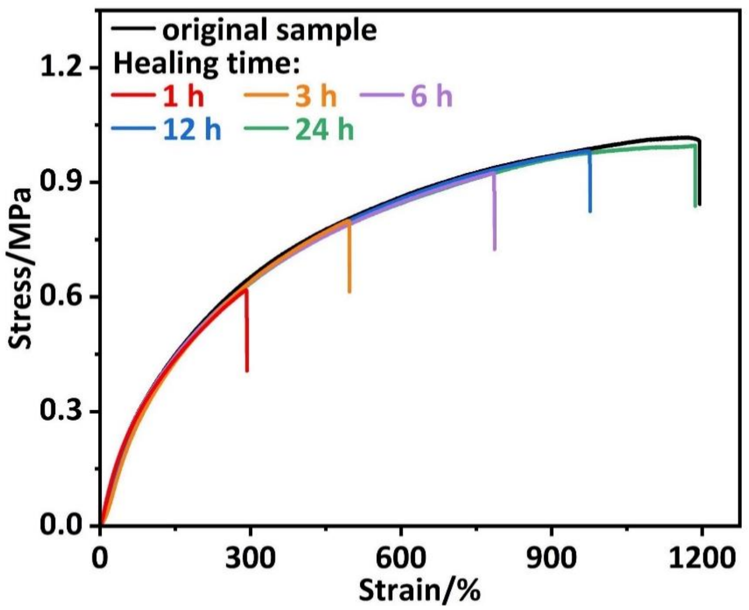

Supplementary Figure 23. Typical stress–strain curves of the original and healed SSPU for different healing time at room temperature. As shown, the healing efficiencies gradually increased with the prolonged healing time. In particular, a prolonged healing time of 24 h resulted in almost fully restoration mechanical properties, including Young's modulus, tensile strength and tensile strain.

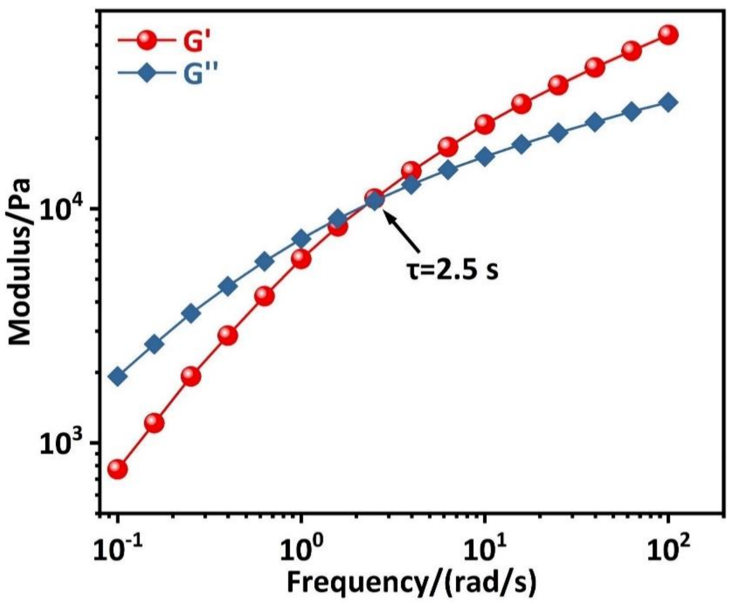

Supplementary Figure 24. Rotational rheology curves of SSPU at 95 °C. As shown, a very short terminal relaxation time was calculated as 2.5 s under a relatively high temperature of 95 °C, which meant that SSPU exhibited very fast self-healing behavior at high temperature.

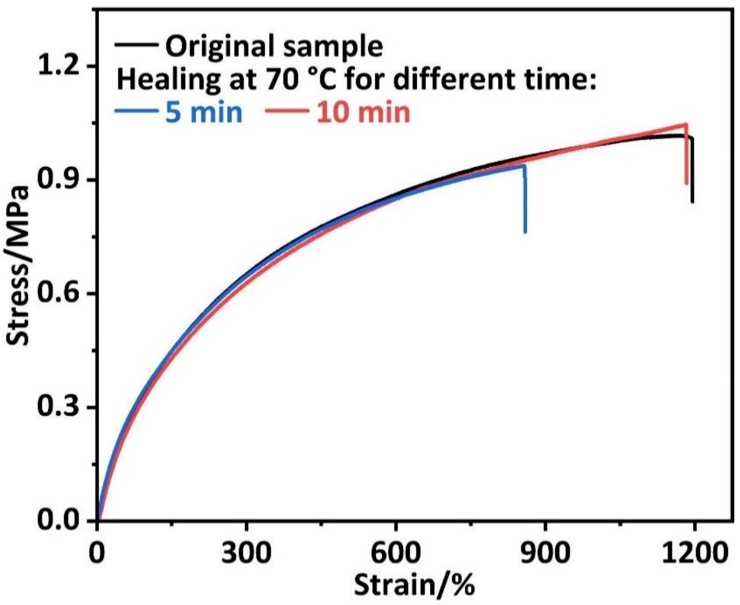

Supplementary Figure 25. Typical stress–strain curves of the original and healed SSPU for different healing time periods at 70 °C. As shown, SSPU could almost completely restore mechanical properties within 10 minutes at 70 °C, which is far superior than self-healing at room temperature.

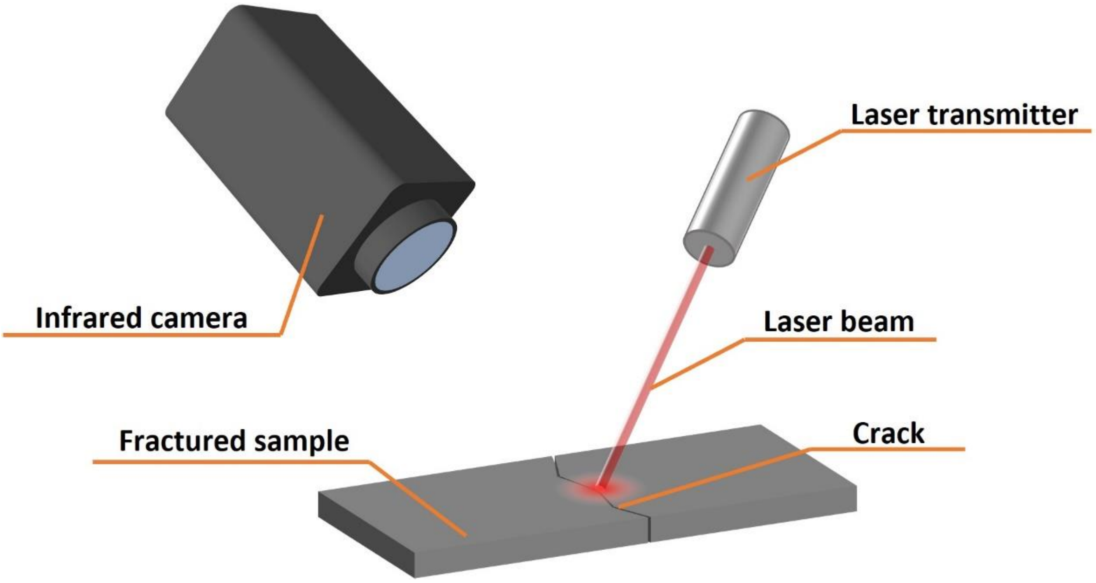

Supplementary Figure 26. Schematic diagram of FLIR camera/laser setup.

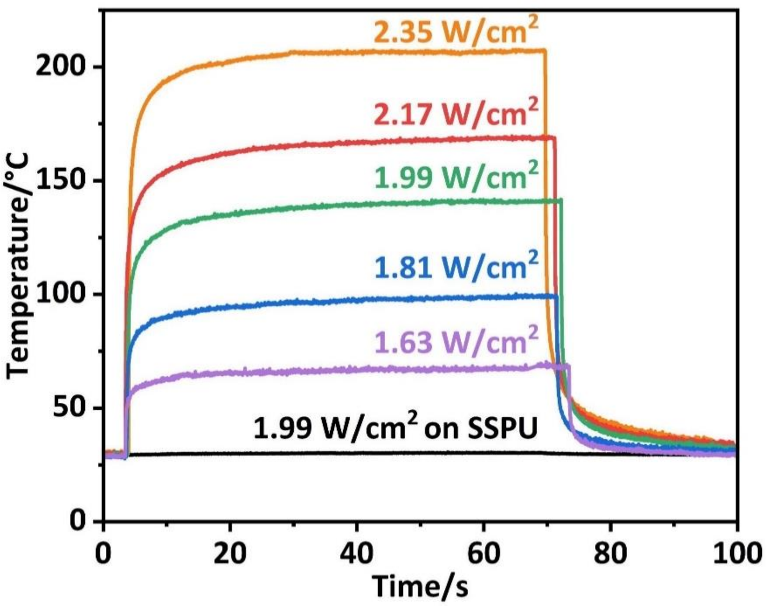

Supplementary Figure 27. Time-dependent photothermal heating curves of varying laser output power for SSPUGIT-3 and SSPU

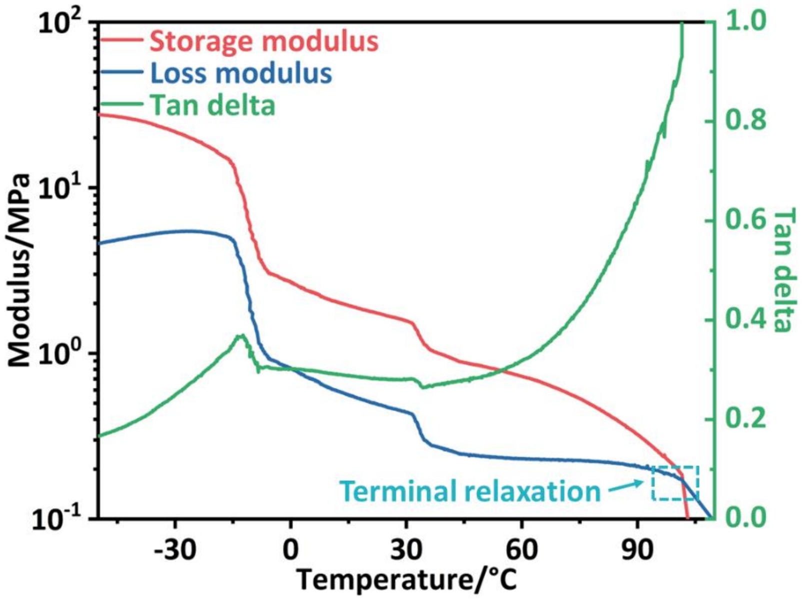

Supplementary Figure 28. Storage modulus, loss modulus and loss factor versus temperature for SSPUGIT-3. As shown, since the terminal relaxation temperature of SSPUGIT-3 was about 101.83 °C, laser output power was controlled to make the polymer chains flow rapidly to induce high-accuracy self-healing. It could be seen that the terminal relaxation temperature of SSPUGIT-3 was a little higher than that of SSPU (Supplementary Fig. 22), which was because the incorporation of Galinstan droplets into SSPU matrix hindered the movement of polymer chains of SSPU to some extent.

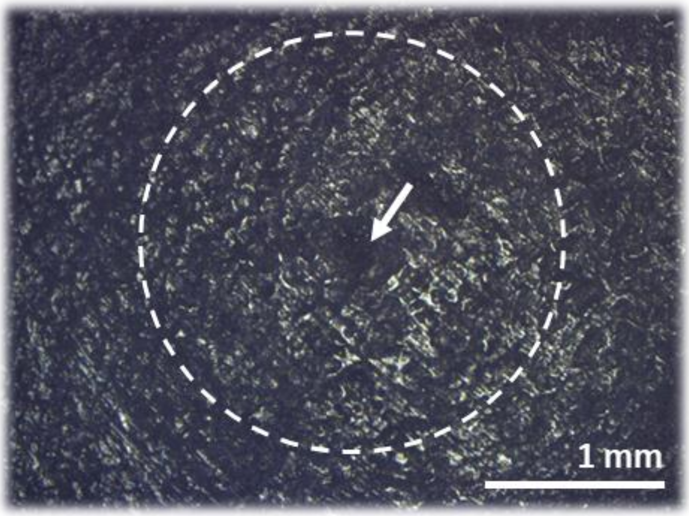

Supplementary Figure 29. Optical microscopy photograph of self-healed SSPUGIT-3 after punctured by a needle.

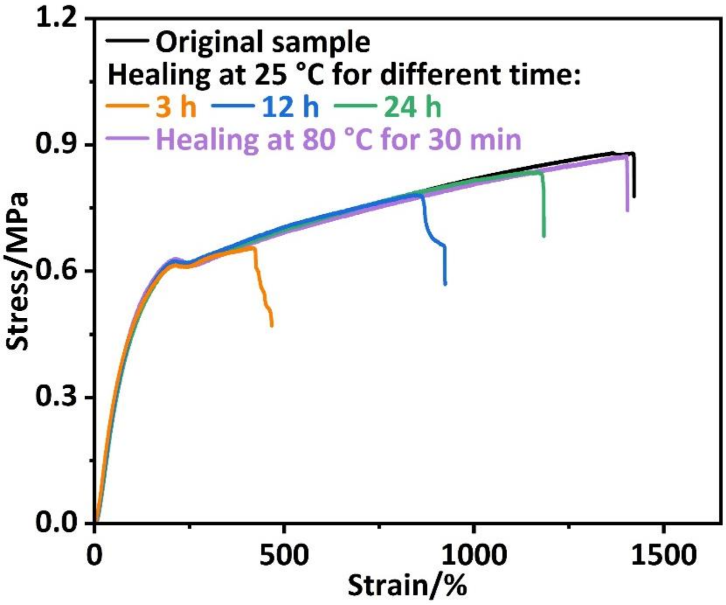

Supplementary Figure 30. Typical stress–strain curves of the original and healed SSPUGIT-3 for different healing time at 25 °C and 80 °C. As shown, the room-temperature self-healing efficiencies of SSPUGIT-3 composite gradually increased with the prolonged healing time. After 24 h of self-healing at 25 °C, SSPUGIT-3 composite could not fully restore its original mechanical properties. Increase of healing temperature of 80 °C can enable a higher self-healing rate and efficiency, i.e., the cut-damaged SSPUGIT-3 almost fully restored its mechanical properties within 30 min, far superior than self-healing at 25 °C.

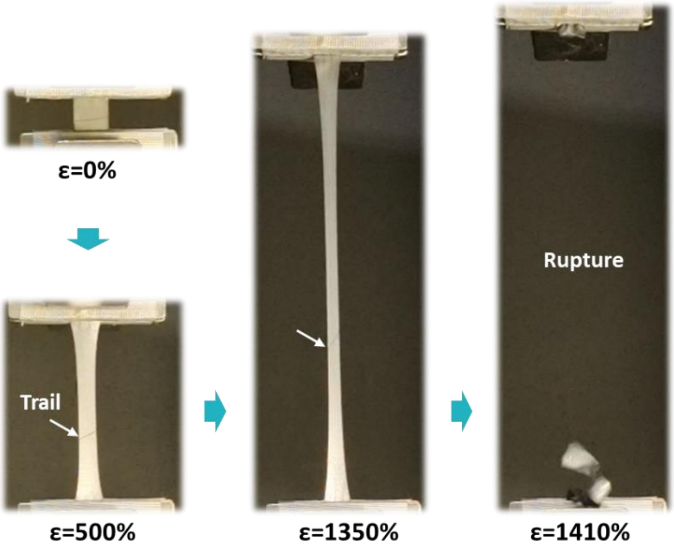

Supplementary Figure 31. Photographs demonstrating the self-healing behavior of SSPUGIT-3 composite under NIR laser. The cut SSPUGIT-3 composite could be brought into contact to induce self-healing at room temperature, but it took long time (>24 h) to complete the healing process. In a sharp contrast, NIR-induced self-healing could be quickly achieved with high accuracy and efficiency. As shown in Supplementary Fig. 31, a visible healing trail was marked in the lower half of the specimen, while this self-healed specimen was ruptured in its upper half part upon stretching, which demonstrated the excellent self-healing capability of SSPUGIT-3 under NIR laser.

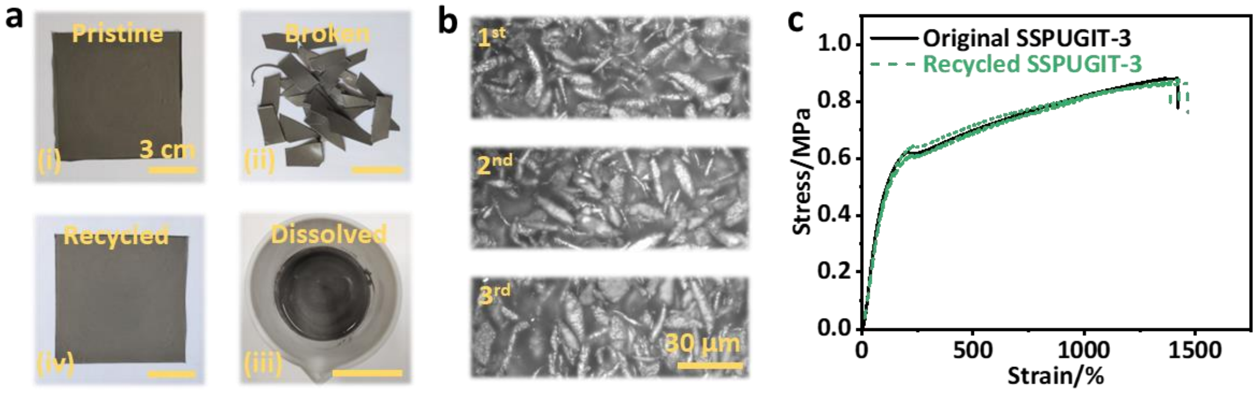

Supplementary Figure 32. (a) Photographs showing the solvent-reprocessability of SSPUGIT-3 composite. (b) Optical microscopy images of the recycled SSPUGIT-3 composites. (c) Stress-strain curves of the original, and recycled SSPUGIT-3 composites. The dynamic and thermoplastic nature of SSPU matrix coupled with the liquid nature of Galinstan droplets enabled the reprocessability of SSPUGIT composites. As shown in Supplementary Fig. 32a, the as-made sheet was first cut into pieces and then rapidly dissolved by trichloromethane in a shear mixer. Such processes resulted in a SSPU and trichloromethane solution with dispersed Galinstan droplets, which was similar to the original composite system. Subsequently, this composite dispersion could be cast into a new sheet via a solvent evaporation method. Satisfactorily, the microstructures of the remolded composites were almost constant even after three cycles (Supplementary Fig. 32b; Fig. 1e). But more than that, the mechanical properties of the recycled composite were also basically maintained at the original levels (Supplementary Fig. 32c), demonstrating the potential for reusing the composite.

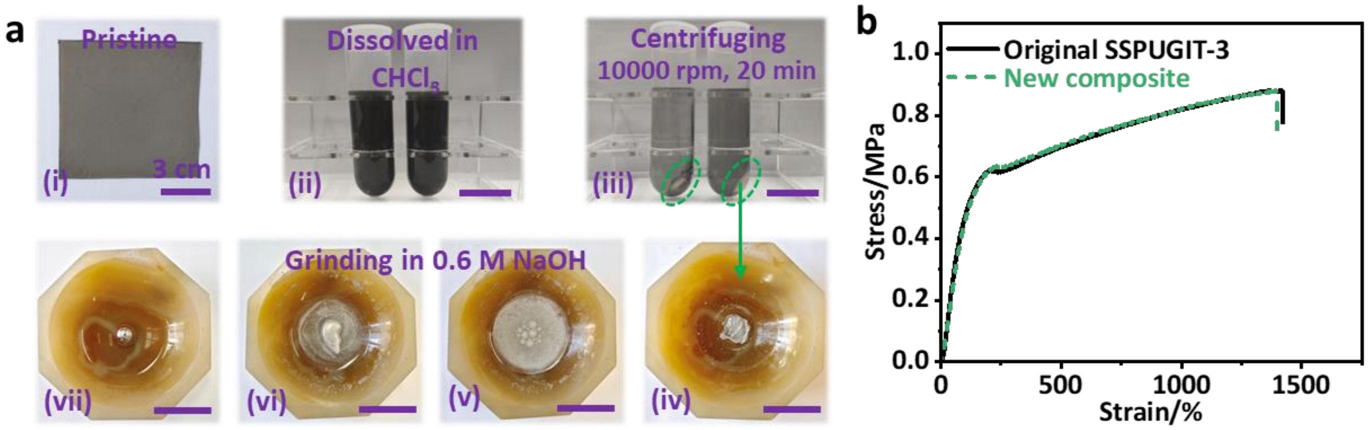

Supplementary Figure 33. (a) Photographs of the recovery process of liquid Galinstan alloys from SSPUGIT-3 composite. (b) Stress-strain curves of the original and new composite fabricated via the recycled Galinstan droplets. As depicted in Supplementary Fig. 33a, SSPUGIT-3 composite was dissolved in $CHCl_3$, which was then centrifuged to obtain Galinstan droplets and SSPU-loaded $CHCl_3$ solution. Noting that the centrifuge effluent consisted of micro- and nanodroplets wrapped in layers of gallium oxide. To achieve a macroscopic Galinstan droplets, base (i.e., 0.6 M NaOH) was employed to remove the oxide layer[16]. As a result, the gathered minuscule droplets were driven to merge into a macroscopic and reusable Galinstan droplets by their high interfacial tension, together with a high recovery efficiency of 84±2%[17]. Intriguingly, the recycled Galinstan droplets could be reused to fabricate new composite, which possessed similar tensile curves as the original composite (Supplementary Fig. 33b).

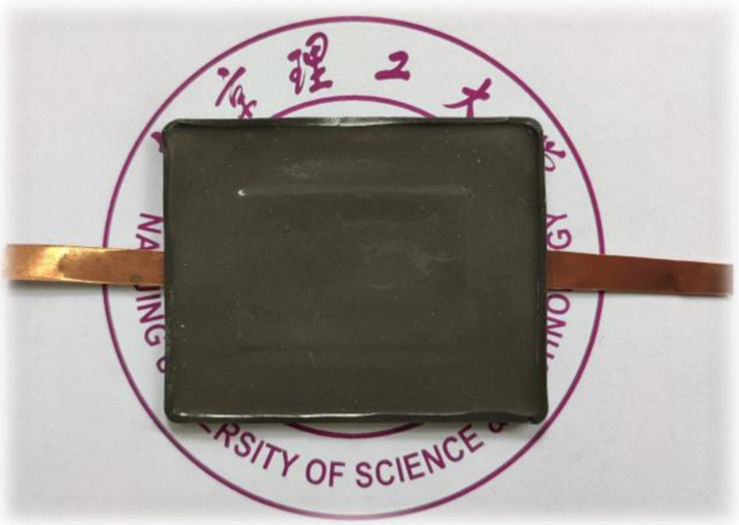

Supplementary Figure 34. Photograph of soft capacitive strain-sensor based on SSPUGIT-3.

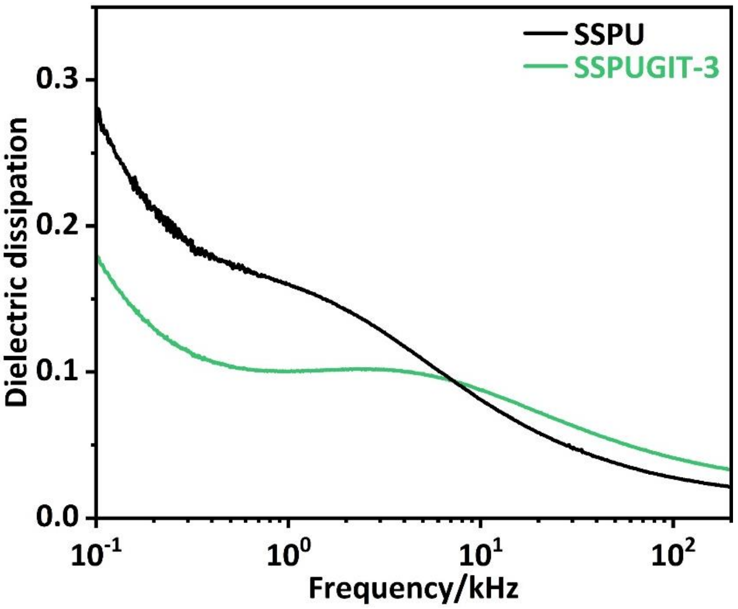

Supplementary Figure 35. Dielectric dissipation of SSPU and SSPUGIT-3 as a function of frequency at 25 °C.

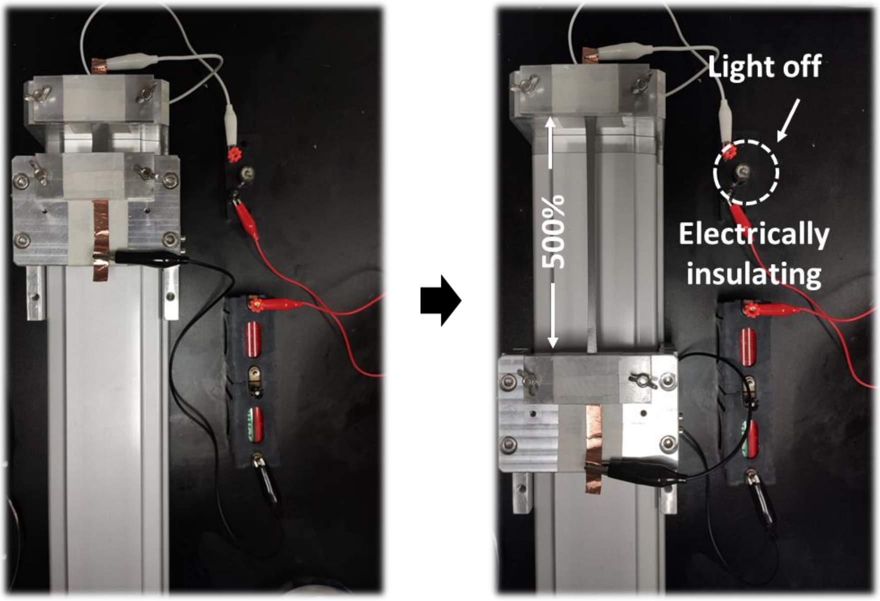

Supplementary Figure 36. Photograph of original and stretched SSPUGIT-3 connected in circuit with light bulb. As shown, SSPUGIT-3 remained electrically insulating even stretched to 500% strain, which was due to the robust polymer interface between individual isolated Galinstan droplet (Figure 3i). For this reason, although the Ga_2O_3 shell of Galinstan droplet ruptured under large strain (>200%), their electrical inner could not fuse together to make SSPUGIT-3 conductive. In general, such a dielectric characteristic of SSPUGIT was mainly attribute to the unique vascular smooth muscle-inspired architecture, which is totally different from other electrically composite based on Galinstan[18-20].

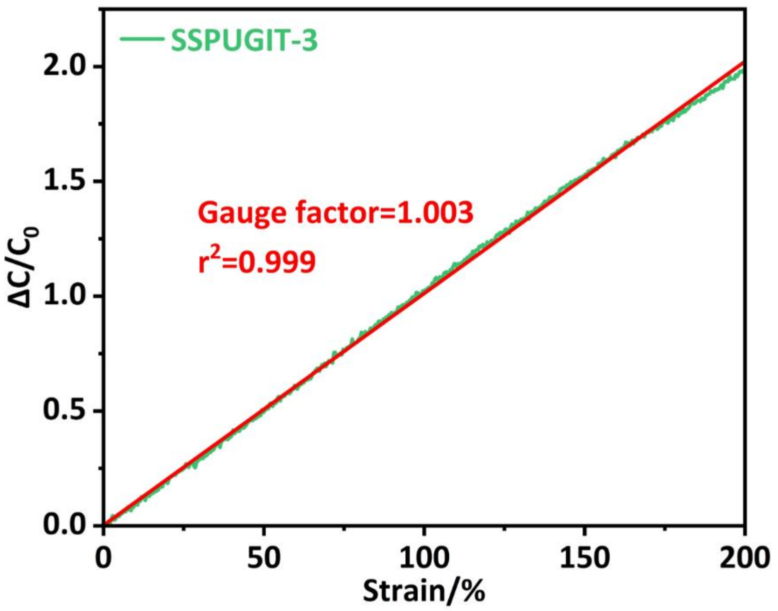

Supplementary Figure 37. Relative capacitance variations of SSPUGIT-3-based capacitive strain-sensors as a function of tensile strain.

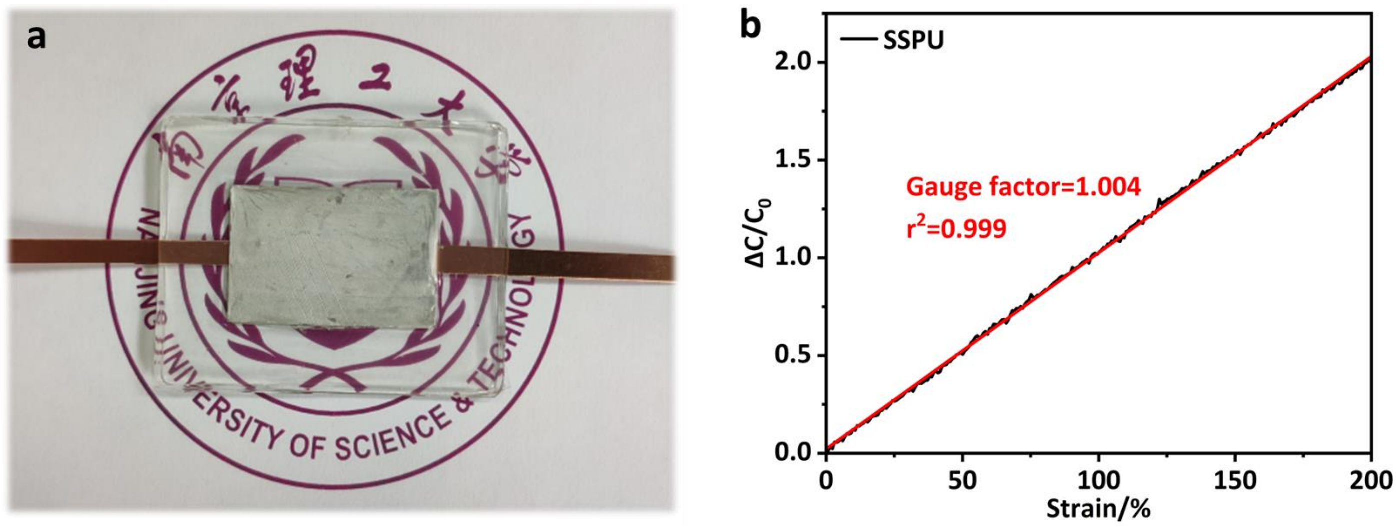

Supplementary Figure 38. (a) Photograph of soft capacitive strain-sensor based on SSPU. (b) Relative capacitance variations of SSPU-based capacitive strain-sensors as a function of tensile strain.

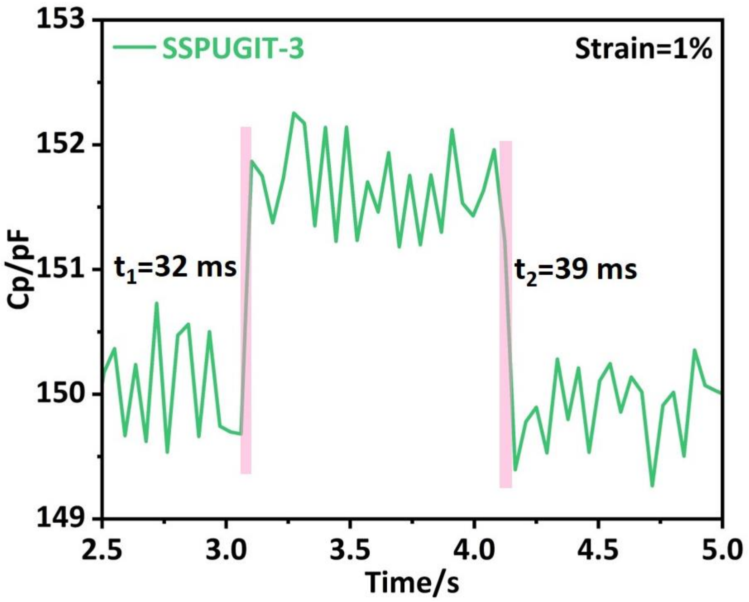

Supplementary Figure 39. Response time of SSPUGIT-3-based capacitive strain-sensor. As shown, the response time of the capacitive strain-sensor was measured by stretching it to 1% strain for 1 s and then relaxing. The response and recovery time for the stretching/relaxing process were 32 ms and 39 ms, respectively.

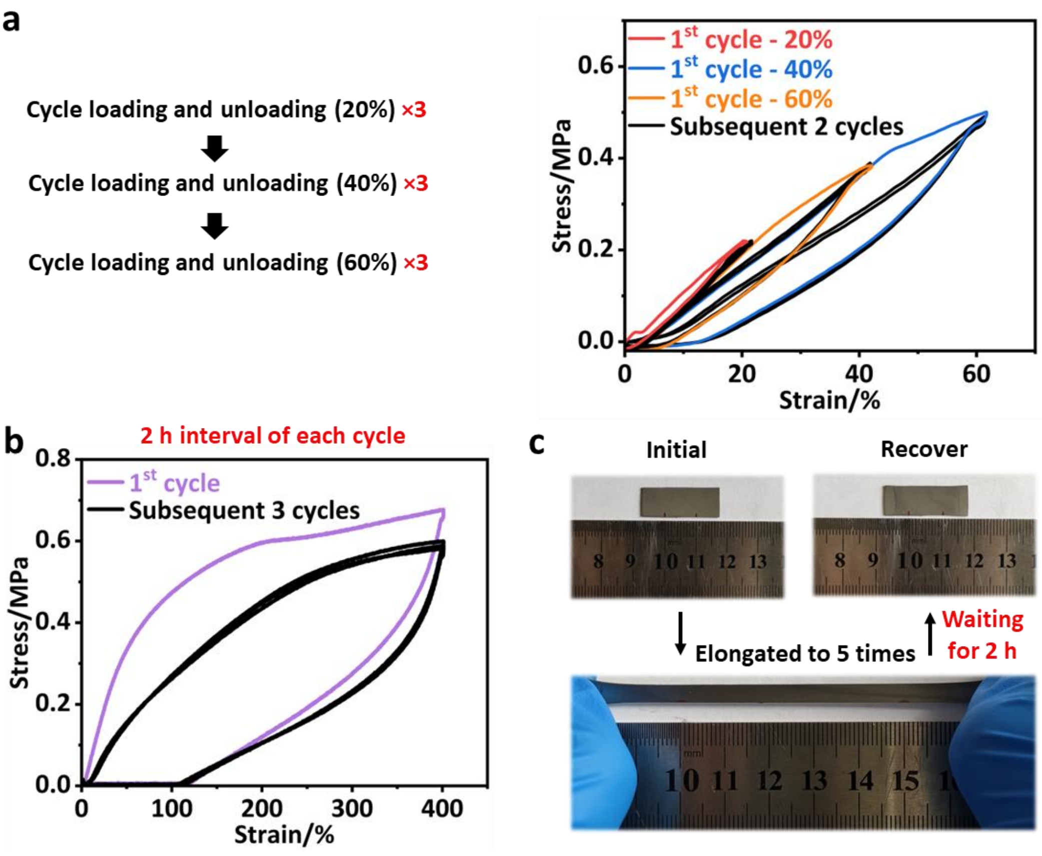

Supplementary Figure 40. (a) Cyclic stress-strain curve of SSPUGIT-3 in successive stretching (20%, 40%, 60% strain), which was repeated three times at each strain. (b) Cyclic stress-strain curve of SSPUGIT-3 under 400% strain, the interval of each cycle was 2 h. (c) Photos of SSPUGIT-3 stretched

to 400% and recovering in 2 h. Following an initial loading cycle, the second loading cycle of SSPUGIT-3 was basically identical to the third cycles with small hysteresis at low strains (<60%) (Supplementary Fig. 40a), demonstrating the fast and elastic deformation behavior, which is highly desired for application as capacitive strain-sensors. Indeed, the difference in the initial loading cycle was attributed to the Mullin's effect[21], which could be observed each time upon the specimen was stretched to a new strain exceeding the previous maximum strain. When the specimen was loaded to a high strain region of 400%, a large hysteresis was observed at the initial loading cycle (Supplementary Fig. 40b), which was not only ascribed to the influence of Mullin's effect, but also due to the breakage of weak hydrogen bonds within dynamic hard domains of SSPU to dissipate strain energy. Notably, the loading and unloading curves of subsequent cycle almost overlapped after being stretched to 400%, suggesting that the hydrogen bonds could completely reform in the relaxation time of 2 h (Supplementary Fig. 40b). Such an excellent recovery ability was also depicted in Supplementary Fig. 40c, in which the elongated SSPUGIT-3 was observed to fully return its original dimension without residual strain after relaxation for 2 h.

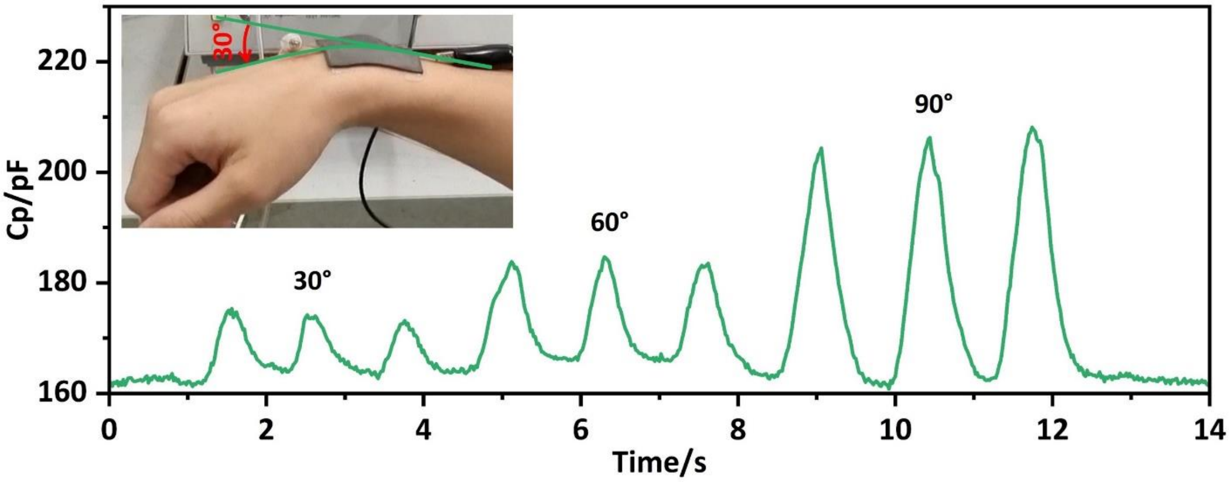

Supplementary Figure 41. Capacitance change of the strain-sensor responds to the wrist bending under different angles. Our SSPUGIT-3-based capacitive strain-sensor showed viscoelastic behavior derived from its polymer part crosslinked by multiple hydrogen bonds. However, as confirmed by cyclic tensile test (Supplementary Fig. 40a), the deformation recovery of SSPUGIT-3 is fast and elastic with small hysteresis at low strain (<60%), making it an ideal candidate for wearable capacitive strain-sensor. Specifically, when this strain-sensor was conformally laminated onto elastic substrates such as finger or wrist, it showed clear, sensitive and stable signals during cyclic human motion detection without any rest (Supplementary Fig. 41).

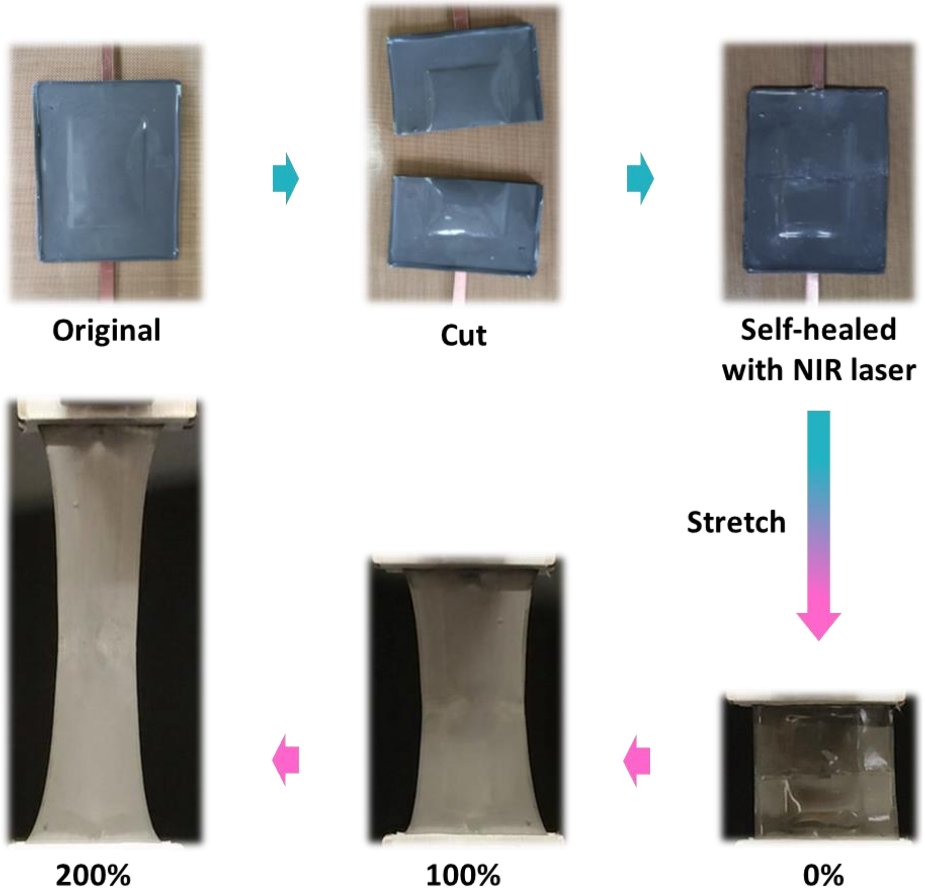

Supplementary Figure 42. Photographs showing the self-healing of mechanical properties of SSPUGIT-3-based strain-sensor under NIR laser

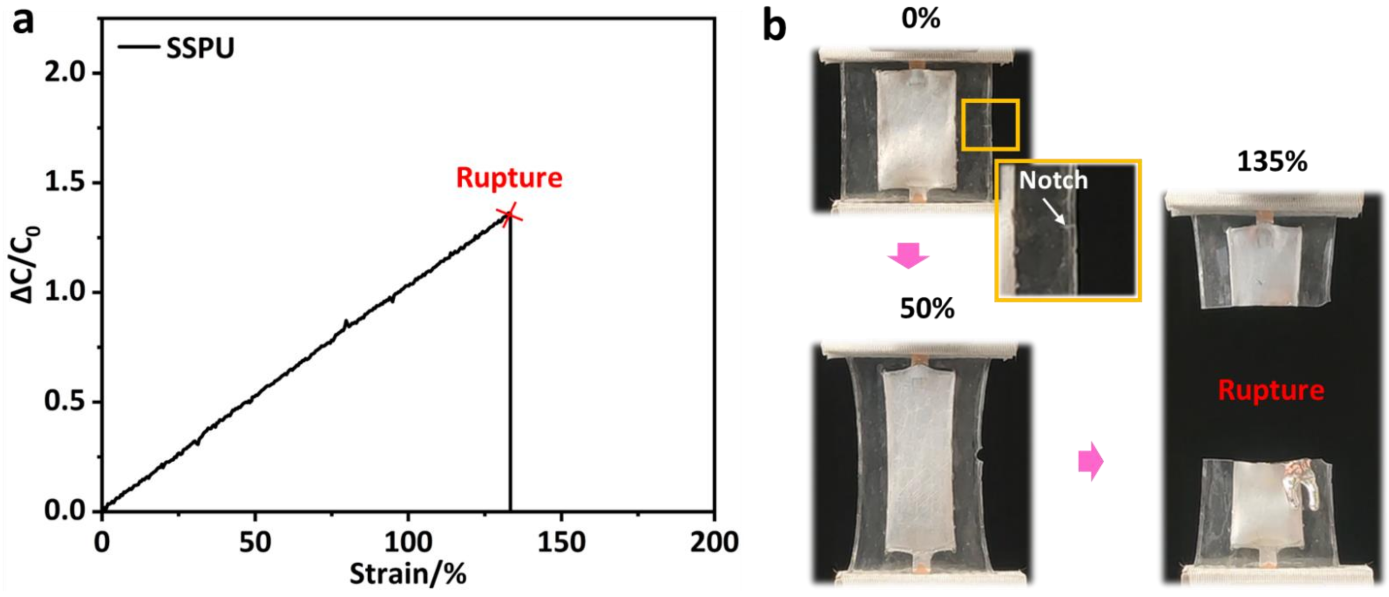

Supplementary Figure 43. (a) Relative capacitance variation of notched SSPUGIT-3-based capacitive strain-sensors as a function of tensile strain to 135%. (b) Optical images of a notched SSPUGIT-3-based capacitive strain-sensor upon stretching to 135%.

Supplementary Table 1. Comparison of the strain retentions of SSPUGIT-3 with state-of-the-art crack-resistant self-healing materials

Sample	Strain retention/%	Suppl. Ref.
BNNSs-LM/PUU	24.22	22
SPM-2	37.50	23
PDMS-MPU-IU	44.96	24
P(NaSS-co-MPTC)	57.78	25
Fe-Hpdca-PDMS	61.28	26
SNPP	75.00	27
SSPUGIT-3	100	Our work

Supplementary Table 2. Comparison of the fracture energy and Young's modulus among state-of-the-art self-healing materials with SSPUGIT-3

Sample	Young's modulus MPa	Fracture energy kJ/m^2	Suppl. Ref.
MEG2-Li	20.30	95.27	28
$G10/T1_{1.6}$	59.40	24.70	29
TFE-HF-$QD_{1.0}$	29.00	30.00	30
$MPM_{0.2}$-Fe	7.00	24.00	2
P(HFBM-co-SBMA)	43.00	15.07	31
TTD_{57}-$IPDA_{43}$	7.50	17.30	32
P(VI-co-AAc)	4.80	5.60	33
P(Nass-co-MPTC)	2.20	4.00	25
B-DN3	2.20	2.85	34
BIIR-PαP-30	4.68	1.50	35
PDMS-MPU-IU	0.62	11.48	24
PR-PDMS-2	0.69	6.77	36
PAAm	1.46	3.79	1
Fe-Hpdca-PDMS	0.54	2.57	26
P-Cur-Eu	0.50	2.44	37
SSPU	0.72	3.18	Our work
SSPUGIT-3	0.83	111.16	

Supplementary Table 3. Comparison of the healing time and healed fracture energy among state-of-the-art soft self-healing materials with SSPUGIT-3

Sample	Healing time min	Healed Fracture energy kJ/m^2	Suppl. Ref.
PCL/PTHF	420	43.30	38
PDA-PNAGA-GO	10	6.99	39
PDA-pGO-PAM	2.5	6.78	40
DN-PDMS-0.5	60	2.90	41
DT-$IPDI_2$-$HMDI_1$	360	7.46	42
H2D4S4	600	13.00	43
PMPTC/PNaSS	720	8.60	44
PUIP-NAGA2.6	360	27.00	45
PDO-IP2.5	720	83.90	46
TTD_{57}-$IPDA_{43}$	1440	17.30	33
SSPUGIT-3	≤1	111.16	Our work

Supplementary Table 4. Comparison of the dielectric permittivity and Young's modulus among commercial dielectric materials with SSPUGIT-3

Sample	Young's modulus MPa	Dielectric permittivity	Suppl. Ref.
PVDF-HFP (Aldrich)	335.00	15.2	47
PVDF (Aldrich)	171.00	10.00	47
PEO (Aldrich)	82.80	5.42	47
Polyurethane (DT6100s)	17.00	7.00	48
Acrylic (3M VHB 4910)	2.30	4.20	49
IPN (VHB 4905-TMPTMA)	3.94	2.43	50
IPN (VHB 4910-TMPTMA)	4.15	3.27	50
Silicone (Dow Sylgard 184)	1.52	3.11	51
SSPU	0.72	4.49	Our work
SSPUGIT-3	0.83	14.57	

Supplementary Table 5. Comparison of variations of the fracture energy and Young's modulus among other toughened materials with SSPUGIT-3

**Sample**	**Young's modulus MPa**	**Fracture energy kJ/m^2**	**Suppl. Ref.**
pectin–Fe^{3+}/PAAm	0.30 to 1.46	0.77 to 3.79	1
MPM-Fe	1.60 to 7.00	3.60 to 24.00	2
PU-BN	1.40 to 3.10	18.80 to 72.10	3
G10/T1	6.50 to 59.40	15.90 to 24.70	4
SSPUGIT	0.72 to 0.83	3.18 to 111.16	Our work

Supplementary References

1. Niu, R. et al. Hybrid pectin–Fe^{3+}/polyacrylamide double network hydrogels with excellent strength, high stiffness, superior toughness and notch-insensitivity. *Soft Matter* **13**, 9237-9245 (2017).
2. Zhao, P. et al. Mussel cuticle-mimetic ultra-tough, self-healing elastomers with double-locked nanodomains exhibit fast stimuli-responsive shape transformation. *J. Mater. Chem. A* **8**, 12463-12471 (2020).
3. Song, K. et al. Synergy between dynamic covalent boronic ester and boron–nitrogen coordination: strategy for self-healing polyurethane elastomers at room temperature with unprecedented mechanical properties. *Mater. Horiz.* **8**, 216-223 (2021).
4. Zhang, H. J. et al. Dynamic bonds enable high toughness and multifunctionality in gelatin/tannic acid-based hydrogels with tunable mechanical properties. *Soft Matter* **17**, 9399-9409 (2021).
5. Sun, F. Y. et. al. An autonomously ultrafast self-healing, highly colourless, tear-resistant and compliant elastomer tailored for transparent electromagnetic interference shielding films integrated in flexible and optical electronics. *Mater. Horiz.* **8**, 3356-3367 (2021).
6. Xu, J. H., Chen, J. Y., Zhang, Y. N., Liu, T. & Fu, J. J. A fast room-temperature self-healing glassy polyurethane. *Angew. Chem., Int. Ed.* **60**, 7947-7955 (2021).
7. Xu, J. H., Ye, S., Ding, C. D., Tan, L. H. & Fu, J. J. Autonomous self-healing supramolecular elastomer reinforced and toughened by graphitic carbon nitride nanosheets tailored for smart anticorrosion coating applications. *J. Mater. Chem. A* **6**, 5887-5898 (2018).
8. Hwang, S. H, Kang, D., Ruoff, R. S., Shin, H. S. & Park, Y. B. Poly(vinyl alcohol) reinforced and toughened with poly(dopamine)-treated graphene oxide, and its use for humidity sensing. *ACS Nano* **8**, 6739-6747 (2014).
9. Wang, Y. et. al. Hierarchically structured self-healing actuators with superfast light- and magnetic-response. *Adv. Funct. Mater.* **29**, 1906198 (2019).
10. Xin, Y., Peng, H., Xu, J. & Zhang, J. Ultrauniform embedded liquid metal in sulfur polymers for recyclable, conductive, and self-healable materials. *Adv. Funct. Mater.* **29**, 1808989 (2019).
11. Ducrot, E., Chen, Y., Bulters, M., Sijbesma, R. P. & Certon, C. Toughening elastomers with sacrificial bonds and watching them break. *Science* **344**, 186-189 (2014).
12. Garboczi, E. J. Linear dielectric-breakdown electrostatics. *Phys. Rev. B* **38**, 9005 (1988).
13. Chen, C., Wang, Z & Suo, Z. Flaw sensitivity of highly stretchable materials. *Extreme Mech. Lett.* **10**, 50-57 (2017).
14. Zhao, J. et. al. Mechanically interlocked vitrimers. *J. Am. Chem. Soc.* **144**, 872-882 (2022).
15. Wang, D. et. al. Transparent, mechanically strong, extremely tough, self-recoverable, healable supramolecular elastomers facilely fabricated via dynamic hard domains design for multifunctional applications. *Adv. Funct. Mater.* **30**, 1907109 (2020).
16. Chen, Y. et. al. Robust fabrication of nonstick, noncorrosive, conductive graphene-coated liquid metal droplets for droplet-based, floating electrodes. *Adv. Funct. Mater.* **28**, 1706277 (2018).
17. Li, F. et. al. Recyclable liquid metal-based circuit on paper. *Adv. Mater. Technol.* **3**, 1800131 (2018).

18. Tang, S. Y. & Qiao R. Liquid metal particles and polymers: A soft−soft system with exciting properties. *Acc. Mater. Rec.* **2**, 966-978 (2021).
19. Park, J. E., Kang, H. S., Koo, M. & Park C. Autonomous surface reconciliation of a liquid-metal conductor micropatterned on a deformable hydrogel. *Adv. Mater*. **32**, 2002178 (2020).
20. Thrasher, C. J., Farrell, Z. J., Morris, N. J., Willey, C, L. & Tabor, C. E. Mechanoresponsive polymerized liquid metal networks. *Adv. Mater.* **31**, 1903864 (2019).
21. Mullins, L. Softening of rubber by deformation. *Rubber Chem. Technol.* **42**, 339 (1969).
22. Wang, D., Liu, D., Xu, J., Fu, J. & Wu, K. Highly thermoconductive yet ultraflexible polymer composites with superior mechanical properties and autonomous self-healing functionality via a binary filler strategy. *Mater. Horiz.* **9**, 640-652 (2022).
23. Yan, X. et al. Quadruple H-Bonding cross-linked supramolecular polymeric materials as substrates for stretchable, antitearing, and self-healable thin film electrodes. *J. Am. Chem. Soc.* **140**, 5280-5289 (2018).
24. Kang, J. et al. Tough and water-insensitive self-healing elastomer for robust electronic skin. *Adv. Mater.* **30**, 1706846 (2018).
25. Sun, T. et al. Physical hydrogels composed of polyampholytes demonstrate high toughness and viscoelasticity. *Nat. Mater.* **12**, 932–937 (2013).
26. Li, C. H. et al. A highly stretchable autonomous self-healing elastomer. *Nat. Chem.* **8**, 618–624 (2016)
27. Qin, H., Zhang, T., Li, N., Cong, H. P. & Yu, S. H. Anisotropic and self-healing hydrogels with multi-responsive actuating capability. *Nat. Commun.* **10**, 2202 (2019).
28. Li, M. et al. Superstretchable, yet stiff, fatigue-resistant ligament-like elastomers. *Nat. Commun.* **13**, 2279 (2022).
29. Zhang, H. J. et al. Dynamic bonds enable high toughness and multifunctionality in gelatin/tannic acid-based hydrogels with tunable mechanical properties. *Soft Matter* **17**, 9399-9409 (2021).
30. Liu, Y. et al. Tough, stable and self-healing luminescent perovskite-polymer matrix applicable to all harsh aquatic environments. *Nat. Commun.* **13**, 1338 (2022).
31. Hu, P. et al. Printable, room-temperature self-healing and full-color-tunable emissive composites for transparent panchromatic display and flexible high-level anti-counterfeiting. *Chem. Eng. J.* **431**, 133728 (2022).
32. Wu, P. et al. New kind of thermoplastic polyurea elastomers synthesized from CO_2 and with self-healing properties. *ACS Sustain. Chem. Eng.* **8**, 12677-12685 (2020).
33. Ding, H. et al. Hydrogen bond reinforced poly(1-vinylimidazole-co-acrylic acid) hydrogels with high toughness, fast self-recovery, and dual PH-responsiveness. *Polymer* **131**, 95-103 (2017).
34. Zhang, H. J. et al. Tough physical double-network hydrogels based on amphiphilic triblock copolymers. *Adv. Mater.* **28**, 4884-4890 (2016).
35. Song, S., Hou, H., Wang, J., Rao, P. & Zhang, Y. A self-healable, stretchable, tear-resistant and sticky elastomer enabled by a facile polymer blends strategy. *J. Mater. Chem. A* **9**, 3931-3939 (2021).
36. Du, R. et al. A highly stretchable and self-healing supramolecular elastomer based on sliding crosslinks and hydrogen bonds. *Adv. Funct. Mater.* **30**, 1907139 (2020).

37. Zhang, Q. et al. An elastic autonomous self-healing capacitive sensor based on a dynamic dual crosslinked chemical system. *Adv. Mater.* **30**, 1801435 (2018).
38. Xun, X. et al. Tough and degradable self-healing elastomer from synergistic soft–hard segments design for biomechano-robust artificial skin. *ACS Nano* **15**, 20656-20665 (2021).
39. Feng, Z. et al. Mussel-inspired highly stretchable, tough nanocomposite hydrogel with self-healable and near-infrared actuated performance. *Ind. Eng. Chem. Res.* **59**, 166-174 (2020).
40. Han, L. et al. A Mussel-inspired conductive, self-adhesive, and self-healable tough hydrogel as cell stimulators and implantable bioelectronics. *Small* **13**, 1601916 (2017).
41. Chen, C., Fei, H.-F., Watkins, J. J. & Crosby, A. J. Soft double-network polydimethylsiloxane: fast healing of fracture toughness. *J. Mater. Chem. A* **10**, 11667-11675 (2022).
42. Zhang, L. et al. A highly stretchable, transparent, notch-insensitive self-healing elastomer for coating. *J. Mater. Chem. C* **8**, 2043-2053 (2020).
43. Yang, J. et al. Fatigue-resistant, notch-insensitive zwitterionic polymer hydrogels with high self-healing ability. *ChemPlusChem* **85**, 2158-2165 (2020).
44. Luo, F. et al. Oppositely charged polyelectrolytes form tough, self-healing, and rebuildable hydrogels. *Adv. Mater.* **27**, 2722-2727 (2015).
45. Yao, Y. et al. Multiple h-bonding chain extender-based ultrastiff thermoplastic polyurethanes with autonomous self-healability, solvent-free adhesiveness, and AIE fluorescence. *Adv. Funct. Mater.* **31**, 2006944 (2021).
46. Liu, X. et al. Engineered self-healable elastomer with giant strength and toughness via phase regulation and mechano-responsive self-reinforcing. *Chem. Eng. J.* **410**, 128300 (2021).
47. Lopez, J. et al. Effects of polymer coatings on electrodeposited lithium metal. *J. Am. Chem. Soc.* **140**, 11735-11744 (2018).
48. Pelrine, R. et al. High-field deformation of elastomeric dielectrics for actuators. *Mater. Sci. Eng. C* **11**, 89-100 (2000).
49. Shankar, R., Ghosh, T. K. & Spontak, R. J. Electromechanical response of nanostructured polymer systems with no mechanical pre-strain. *Macromol. Rapid Commun.* **28**, 1142-1147 (2007).
50. Ha, S. M. et al. High electromechanical performance of electroelastomers based on interpenetrating polymer networks. *Proc. SPIE.* **6927**,69272C (2008).
51. Pan, C. et al. A liquid-metal–elastomer nanocomposite for stretchable dielectric materials. *Adv. Mater.* **31**, 1900663 (2019).
