# Supplementary material for: Vascular smooth muscle-inspired architecture enables soft yet tough self-healing materials for durable capacitive strain-sensor
Source: Nat Commun. 2023 Jan 10;14:130. doi: 10.1038/s41467-023-35810-y (PMC9829674; doi:10.1038/s41467-023-35810-y)
Supplement: Supplementary file 2 — Description of Additional Supplementary Files [file 41467_2023_35810_MOESM2_ESM.pdf]

Description of Additional Supplementary Files

File name: Supplementary Movie 1

Description: This movie showed that the notched SSPU could only be stretched to 1.2× its original length at a deformation rate of 100 mm min-1, far inferior to that of intact sample (11.9×). The gauge length, width and crack length were 10 mm, 60 mm and 20 mm, respectively.

File name: Supplementary Movie 2

Description: This movie showed that the notched SSPUGIT-3-C could only be stretched 1.9× its original length at a deformation rate of 100 mm min-1, far inferior to that of intact sample (5.2×). The gauge length, width and crack length were 10 mm, 60 mm and 20 mm, respectively.

File name: Supplementary Movie 3

Description: This movie showed the outstanding crack-resistance of SSPUGIT-3, where both the 1/3- and 2/3-notched samples could be stretched to 15.4× its original length at a deformation rate of 100 mm min-1, which is the same as the intact sample (15.4×). The gauge length and width of the sample were 10 mm, 60 mm,

respectively. The crack length were 20 mm and 40 mm for the 1/3- and 2/3-notched samples, respectively.

File name: Supplementary Movie 4

Description: This movie exhibited that SSPUGIT-3-based strain-sensor showed clear, sensitive and stable signals during cyclic human motion detection.

File name: Supplementary Movie 5

Description: This movie demonstrated that the pre-damaged SSPU-based strain-sensor having a notch of 2 mm would be fractured upon stretched to 135%, far inferior to that of SSPUGIT-3-based strain-sensor which could be successfully stretched to 200% strain without rupture.
